# Comparison of Proteomic Analysis of Cerebrospinal Fluid From Neurological Patients With and Without Amyotrophic Lateral Sclerosis

**DOI:** 10.1111/jnc.70508

**Published:** 2026-06-26

**Authors:** Eleonora Sabetta, Karin Rallmann, Pille Taba, Abigail L. Pfaff, Bal Hari Poudel, Davide Ferrari, Massimo Locatelli, Sulev Kõks, Jonas Bergquist

**Affiliations:** ^1^ IRCCS Ospedale San Raffaele Milan Italy; ^2^ Department of Neurology Tartu University Hospital Tartu Estonia; ^3^ Institute of Clinical Medicine University Tartu Tartu Estonia; ^4^ Perron Institute for Neurological and Translational Science Perth Western Australia Australia; ^5^ Personalised Medicine Center, Murdoch University, Western Australia Murdoch University Perth Western Australia Australia; ^6^ SCVSA Department University of Parma Parma Italy; ^7^ Analytical Chemistry and Neurochemistry, Department of Chemistry for Life Sciences, Biomedical Center Uppsala University Uppsala Sweden; ^8^ The OMF ME/CFS Collaborative Research Center at Uppsala University Biomedical Center Uppsala Sweden

**Keywords:** genetic architecture, neurodegenerative disorders, protein profiling, proteomic biomarkers, sporadic amyotrophic lateral sclerosis (sALS)

## Abstract

Amyotrophic lateral sclerosis (ALS) is a neurodegenerative disorder characterised by progressive muscle weakness in both bulbar and extremity muscles, leading to a diverse clinical phenotype with motor and non‐motor symptoms. Approximately 85% of ALS cases are sporadic (sALS), while the remaining 10%–15% are familial (fALS). Biological biomarkers of sporadic ALS remain poorly understood, hindering precise patient screening, delaying diagnosis and negatively affecting prognosis. This study aims to identify potential proteomic biomarkers by comparing the cerebrospinal fluid (CSF) of sALS patients with that of patients suffering from other neurological diseases. Liquid chromatography–tandem mass spectrometry (LC–MS/MS) was used for proteomic profiling of CSF samples from 24 sALS patients and 26 patients with other neurological diseases. The complete protein expression profiles were compared using a two‐tailed Student's *t*‐test, with a *p* < 0.05 considered statistically significant with additional FDR correction at the 0.1 level. Proteomic analysis of CSF samples identified significant quantitative changes in 96 proteins with threshold *p* < 0.05 and 74 proteins with FDR < 0.1 between sALS and non‐ALS patients, including alterations in proteins associated with neurodegenerative processes, such as amyloid precursor proteins and inflammatory markers. CSF proteomic analysis reveals altered inflammatory and neurodegenerative metabolic pathways, providing valuable insights into the proteomic landscape of sALS. Several dysregulated proteins were consistent with the disease mechanisms highlighted in previous studies. These findings represent a step forward in developing personalised approaches for diagnosing and managing the disease.

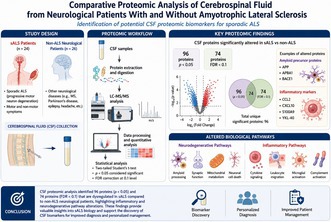

## Graphical abstract

Amyotrophic lateral sclerosis (ALS) is a neurodegenerative disorder characterised by progressive muscle weakness in both bulbar and extremity muscles, leading to a diverse clinical phenotype with motor and non‐motor symptoms. Cerebrospinal fluid (CSF) from sporadic ALS patients were analysed for proteomics content and revealed altered inflammatory and neurodegenerative metabolic pathways as compared to non‐ALS disease controls. These findings represent an important step forward in developing personalised approaches for diagnosing and managing the disease.

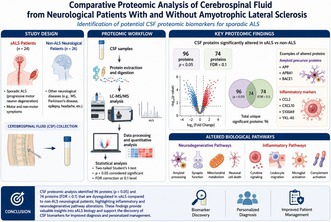

**Abbreviations**
AGC: automatic gain control
ALS: amyotrophic lateral sclerosis
CSF: cerebrospinal fluid
ER: endoplasmatic reticulum
fALS: familial amyotrophic lateral sclerosis
FDR: false discovery rate
FTD: frontotemporal dementia
LC–MS/MS: liquid chromatography–tandem mass spectrometry
PD: Parkinson's disease
sALS: sporadic amyotrophic lateral sclerosis

## 1 Introduction

Amyotrophic lateral sclerosis (ALS) is a neurodegenerative disorder presenting progressive weakness of the bulbar and extremity muscles (Goutman et al. 2022), leading to a wide‐ranging clinical phenotype, that includes both motor and non‐motor symptoms (Urso et al. 2022). Cognitive impairment affects up to 50% of patients (Hardiman and Figlewicz 2012), with 15% diagnosed with frontotemporal dementia (FTD) (Urso et al. 2022). ALS is more common in males than in females (Feldman et al. 2022), with symptoms onset typically between 58 and 63 years (Zarei et al. 2015), and a prevalence of 2.2 per 100 000 individuals in the European population (Logroscino and Piccininni 2019). Approximately 85% of ALS cases are sporadic (sALS), while the remaining 10%–15% are familial (fALS) (Feldman et al. 2022). Both sALS and fALS present similarly (Zarei et al. 2015), with progressive muscle weakness, atrophy, cognitive impairment and executive dysfunction, all influenced by the extent of upper and lower motor neuron involvement and the site of onset (Masrori and Van Damme 2020).

ALS aetiology is influenced by a combination of genetic susceptibility, age‐related cellular damage and environmental exposures (Feldman et al. 2022). The familial form is most commonly associated with mutations in the *SOD1*, *C9orf72*, *FUS* and *TARDBP* genes, accounting for 47.7% of cases, while these variants are present in only a minority of sALS patients (5.2% in the European and Asian populations) (Savage et al. 2019). Monogenic inheritance of rare variants constitutes the basis of fALS. Yet in sALS, there is a growing interest in the effect of oligogenic and polygenic inheritance on the disease risk (Feldman et al. 2022), with some genes possibly exerting a pleiotropic effect on the phenotype (Kirola et al. 2022).

Identifying biomarkers associated with the ALS/FTD spectrum could enable earlier therapeutic interventions, improve outcome prediction and enhance disease monitoring (Vignaroli et al. 2023). Proteomics plays a key role in elucidating ALS pathogenesis by revealing disease‐related alterations in protein expression (Vignaroli et al. 2023). A comprehensive analysis of protein patterns may clarify underlying biochemical mechanisms and assist in distinguishing between subtypes of neurodegenerative dementias (Bibl et al. 2012). Proteomic analysis of cerebrospinal fluid (CSF) has shown potential in characterising modifications in protein expression levels specific to ALS‐FTD, supporting disease progression monitoring and prognostic assessment (Vignaroli et al. 2023).

A comprehensive analysis of the synaptic proteome in ALS revealed shared pathways across different neurodegenerative diseases and potential therapeutic targets, underscoring the central role of the synaptic microenvironment in disease progression (Aly et al. 2023). Characterising neuroinflammatory signatures in both fALS and sALS cohorts could reveal the heterogeneity of inflammatory responses, highlighting their potential as therapeutic targets (Rifai et al. 2023).

Several biomarkers, including genetic, biochemical and imaging‐based markers, have been proposed, but establishing a comprehensive profile of disease‐specific features remains challenging. A recent review summarised proteomic studies in human tissues, plasma, cerebrospinal fluid and exosomes in ALS and Parkinson's disease (PD) that identified proteins with potential utility as biomarkers (Raghunathan et al. 2022). Comparison of proteomic biomarker studies across tissues, biofluids and exosomes in ALS revealed that proteins involved in transcriptional pathways were altered in both spinal cord tissue and the CSF proteome (Bereman et al. 2018; Hartmann et al. 2018; Liu et al. 2022; Oeckl et al. 2020; Engelen‐Lee et al. 2017). In particular, UCHL1, MAP2 and GPNMB were upregulated in spinal cord tissue and CSF in ALS (Bereman et al. 2018). Although proteomics holds substantial clinical potential for biomarker discovery, the number of validated proteomic biomarkers remains limited, owing both to the complexity of the pathology and to the lack of standardised assay validation protocols (Raghunathan et al. 2022). In this study, we aimed to identify CSF proteomic biomarkers by comparing results from a sALS cohort with those of patients diagnosed with other neurological diseases.

## 2 Materials and Methods

### 2.1 Population Characteristics

Between 2013 and 2018, 50 patients from the Neurology Department at the University of Tartu diagnosed with various neurological diseases were included in the study. CSF samples were collected from 24 patients (median and mean age 64.5; SD = 8.87), diagnosed with sALS (ALS‐group) based on El Escorial Criteria and the absence of a positive family history. The most frequent clinical subtype was the classic ALS (83.3%), with spinal symptoms as the most common (62%).

CSF samples were also collected from the remaining 24 patients (median age 64; SD = 11.0) diagnosed with other neurological diseases, different from ALS (non‐ALS‐group), including Bell's paralysis, cognitive dysfunction, diplopia, mononeuritis multiplex, non‐specific white matter lesion in MRT, spinal stroke, stroke, transient ischemic attack and suspicion of CNS infection (non‐confirmed).

No power‐analysis for the sample size was calculated a priori, and instead based on previous studies of a similar nature (Niemela et al. 2021; Tarazona et al. 2020; Chao et al. 2010).

The patient selection process was conducted using hospital records, consultations with neurologists and the Estonian Health Insurance Fund's national health data repository. At the time of analysis we had the following basic clinical chemistry data available: For serum—WBC, Hbc, PLT, CRP, sodium, potassium, urea, creatinine, myoglobin, creatine kinase, aspartate aminotransferase, Alanine aminotransferase, bilirubin, gamma‐glutamyl transferase, total protein, albumin, cholesterol, HDL, LDL and for CSF—CSF pleocytosis, CSF protein, CSF lactate, glucose, IgG, Ig index, oligoclonal band (all were negative). No obvious differences could be detected in these basic clinical chemistry data.

The research was approved by the University of Tartu Research Ethics Committee (approval number: 327/T‐17), and all participants provided written informed consent.

The general characteristics of the population are reported in Table 1.

**TABLE 1:** General features of the population subjected to CSF proteomic analysis.

Subjects			
ALS group overall (*n* = 24: 13 females, 11 males)		Non‐ALS group (*n* = 24: 12 females, 12 males)	
Age. Years females: median (SD)	66.0 (8.83)	Age. Years females: median (SD)	70.5 (9.87)
Age. Years males: median (SD)	64.0 (8.55)	Age. Years males: median (SD)	62.5 (12.0)
Age. Years total: median (SD)	64.5 (8.87)	Age. Years total: median (SD)	64 (11.0)
Age at onset. Years: median (IQR)	62.5 (13)	Ethnicity: Caucasian	100%
Ethnicity: Caucasian	100%	Diagnosis	
Duration of diagnosis, months: median (IQR)	16.0 (14.5)	Bell paralysis	2 (7.7%)
Familial ALS	0%	Cognitive dysfunction	1 (3.8%)
Diagnosis/clinical subtype of ALS		Diplopia	1 (3.8%)
Classic ALS	20 (83.3%)	Mononeuritis multiplex	1 (3.8%)
Progressive Muscular Atrophy	2 (8.3%)	Non‐specific white matter lesion in MRT	9 (34.6%)
Progressive Bulbar Palsy	2 (8.3%)	Spinal stroke	1 (3.8%)
Symptoms at onset		Stroke	4 (15.4%)
Spinal	15 (62%)	Transient ischemic attack	1 (3.8%)
Bulbar	9 (38%)	Suspicion of CNS infection (non‐confirmed)	6 (23.1%)

*Note:* Categorical variables were expressed as absolute count (%), while continuous variables were expressed as median (SD). A statistical analysis (Chi‐squared test) showed no significant differences in sex distribution between the groups (*p* = 1). Age was normally distributed between the diagnostic groups, without a statistical difference between diagnostic groups (*p* = 0.73) and between the sexes of subjects (*p* = 0.94).

### 2.2 Proteomic Analysis

Acetonitrile (ACN), methanol (MeOH), acetic acid (HAc), formic acid (FA), ammonium bicarbonate (NH_4_HCO_3_) were obtained from Merck (Darmstadt, Germany). Acetone, protease inhibitor cocktail, phosphate buffered saline (PBS) and trifluoroacetic acid (TFA) were purchased from Sigma Aldrich (St. Louis, MO). For tryptic digestion, iodoacetamide (IAA), urea and dithiothreitol (DTT) were obtained from Sigma Aldrich and trypsin/Lys‐C mixture (mass spectrometry grade) was obtained from Promega (Mannheim, Germany). Ultrapure water was prepared by Milli‐Q water purification system (Millipore, Bedford, MA).

Proteomic analysis was performed on 50 CSF samples (24 from the ALS group and 26 from the non‐ALS group). A 125 μL aliquot of each CSF sample was filtered through a 0.22 μm cellulose acetate spin filter (Agilent Technologies, CA, USA) by centrifugation at 15 000×*g* for 2 min.

The filtered samples were then depleted using the human Multiple Affinity Removal System (MARS Hu‐14) by loading a 120 μL aliquot onto a MARS Hu‐14 cartridge (Agilent Technologies, CA, USA), and collecting the flow‐through (FT) fractions by centrifugation at 100×*g* for 2 min. To maximise yield, two successive wash steps with 400 μL of MARS‐14 Buffer A were performed. The FT and wash (W) fractions were then combined (FT + W) and dried using a speed‐vac.

Proteins were reduced, alkylated and digested in solution with trypsin following a standard protocol. The proteins in the depleted CSF sample were digested using a trypsin/Lys‐C mixture. Briefly, the proteins were re‐dissolved in 50 μL of digestion buffer (6 M urea, 50 mM NH_4_HCO_3_). Ten microliters of 45 mM aqueous DTT were then added to all samples and the mixtures were incubated at 50°C for 150 min to reduce the disulphide bridges. The samples were cooled to room temperature (RT) and 10 μL of 100 mM aqueous IAA was added before incubating the mixtures for an additional 15 min at RT in darkness to carbamidomethylate the cysteines. Finally, a volume of 50 μL of 100 mM NH_4_HCO_3_ was added to all samples followed by the trypsin/Lys‐C mixture dissolved in 500 mM NH_4_HCO_3_, yielding a final trypsin/protein concentration of 5% (w/w). The tryptic digestion was performed overnight at 37°C. Before MS analysis, the peptides were purified and desalted by Pierce C18 Spin Columns (Thermo Fisher Scientific). These columns were activated by 2 × 200 μL of 50% ACN and equilibrated with 2 × 200 μL of 0.5% TFA. The tryptic peptides were adsorbed to the media using two repeated cycles of 40 μL sample loading and the column was washed using 3 × 200 μL of 0.5% TFA. Finally, the peptides were eluted in 3 × 50 μL of 70% ACN and dried. The dried peptides were reconstituted in 40 μL of 0.1% formic acid and further diluted 3‐fold prior to LC–MS/MS analysis.

The samples were analysed using a QExactive Plus Orbitrap mass spectrometer (Thermo Fisher Scientific, Bremen, Germany) equipped with a nano electrospray ion source. The peptides were separated by reversed phase liquid chromatography using an EASY‐nLC 1000 system (Thermo Fisher Scientific). A set‐up of pre‐column and analytical column was used. The pre‐column was a 2 cm EASY‐column (1D 100 μm, 5 μm C18) (Thermo Fisher Scientific) while the analytical column was a 10 cm EASY‐column (ID 75 μm, 3 μm, C18; Thermo Fisher Scientific). Peptides were eluted with a 150 min linear gradient from 4% to 100% ACN at 250 nL/min flow rate. The mass spectrometer was operated in positive ion mode acquiring a survey mass spectrum with resolving power 70 000 (full width half maximum), m/z = 400–1750 using an automatic gain control (AGC) target of 3 × 10^6^. The 10 most intense ions were selected for higher‐energy collisional dissociation (HCD) fragmentation (25% normalised collision energy) and MS/MS spectra were generated with an AGC target of 5 × 10^5^ at a resolution of 17 500. The mass spectrometer worked in data‐dependent acquisition mode. Quantitation is relative between the samples run together. The instrument is subject to ‘drift’ during an experiment, which can cause false differences if, for instance, samples belonging to the same group are run in a sequence. The sample run order was planned to avoid this type of bias.

### 2.3 Qualitative and Quantitative Proteomic Analysis

The acquired data (.RAW‐files) were processed in the MaxQuant software (version 1.5.1.2) and database searches were performed using the implemented Andromeda search engine. MS/MS spectra were correlated to a FASTA database containing proteins from *Homo sapiens* extracted from the Uniprot database (release June 2021). A decoy search database, including common contaminants and a reverse database, was used to estimate the identification false discovery rate (FDR). An FDR of 1% was accepted. The search parameters included: maximum 10 ppm and 0.6 Da error tolerances for the survey scan and MS/MS analysis, respectively; enzyme specificity was trypsin; maximum one missed cleavage site allowed; cysteine carbamidomethylation was set as static modification and oxidation (M), and deamidation of asparagine/glutamine as variable modifications were set as variable modifications. The search criteria for protein identification were set to at least two matching peptides.

Label free quantification was applied for comparative proteomics. All samples were included in the quantitative analysis. Protein identification was performed by a search against the same database as for the qualitative analysis. Results from all samples were integrated to generate total label‐free intensity values per sample. A two‐tailed Student's *t*‐test was applied to the complete protein expression dataset, with statistical significance set at *p* < 0.05. Nominal *p*‐values were corrected with a false discovery rate (FDR) adjustment and a threshold of 0.1. Two comparative analyses were conducted: one included only proteins identified in all 50 samples (called ‘no‐missing values’ throughout the paper), while the second considered proteins detected in at least 80% of the samples (called ‘20%‐missing values’ throughout the paper), specifically, in at least 21 of 26 ALS samples and 19 of 24 non‐ALS samples. Proteomic abundance data have been log‐transformed to approximate a normal distribution and allow for better representation in the figures included.

### 2.4 Functional Annotation and Enrichment Analysis

Functional annotation and semantic similarity analysis were performed using a combination of R packages: ReactomePA (Reactome pathway enrichment), DOSE (Disease Ontology semantic similarity), and clusterProfiler (KEGG pathway annotation). ReactomePA was used for the initial enrichment (overrepresentation) analysis, a standard approach to uncover shared biological themes among differentially expressed proteins or genes. These tools use hypergeometric tests to assess whether the proportion of proteins associated with a given pathway is significantly higher than expected by chance. The analysis focused on identifying enriched pathways within the proteomic dataset. ReactomePA, DOSE and clusterProfiler offer complementary approaches to interpreting human Entrez gene lists. ReactomePA conducts overrepresentation analysis (ORA) using curated Reactome pathway gene sets (total 2848 pathways), assessing whether pathway members are more frequently represented in the list than expected by chance, with multiple‐testing correction. ClusterProfiler performs similar ORA with KEGG 314 human‐specific pathway data, facilitating pathway ranking and visualisation, and emphasising biological themes at the pathway level. DOSE supports Disease Ontology analysis through (i) ORA of DO terms and (ii) semantic similarity, which leverages the DO graph and information content to measure relatedness among 11 000 disease terms and minimise redundancy. All these tools report *p*‐values and FDR‐adjusted *p*‐values, which are all presented in the tables describing the results.

## 3 Results

### 3.1 Proteome Overview and Differential Expression Analysis

A total of 1223 non‐redundant proteins were identified across the 50 cerebrospinal fluid (CSF) samples. To ensure robustness of quantification, two complementary filtering strategies were applied based on missingness thresholds.

First, proteins detected in at least 80% of samples (≤ 20% missing values) were retained for quantitative analysis, yielding 348 reliably quantified proteins. Within this dataset, 94 proteins showed differential expression at *p* < 0.05, of which 74 remained significant after false discovery rate (FDR) correction (FDR < 0.1). Of these, 59 proteins were upregulated and 15 were downregulated in the ALS group (Table S1, Figure S1).

Second, a more stringent analysis restricted to proteins quantified in all 50 samples (no missing values) identified 186 proteins. Within this subset, 58 proteins were significantly altered at *p* < 0.05, and 48 remained significant after FDR correction (FDR < 0.1), including 39 upregulated and 9 downregulated proteins in ALS (Table S2, Figure S2).

Across both analytical strategies, effect sizes were moderate but consistent. Upregulated proteins showed fold‐changes ranging from 1.12 to 1.66 (20% missingness dataset) and 1.16 to 1.66 (complete‐case dataset), while downregulated proteins ranged from 0.83 to 0.42 across both analyses.

Overall, the data revealed a reproducible proteomic signature in ALS CSF characterised by a predominance of upregulated proteins and coherent directional changes across filtering strategies. CVs for all proteins are presented in Table S3A–D.

### 3.2 Functional Enrichment and Pathway‐Level Organisation of the CSF Proteome

Functional enrichment analysis revealed strong convergence across ReactomePA, KEGG and DOSE databases, indicating coordinated dysregulation of immune, proteostatic and extracellular matrix‐related pathways in ALS CSF.

**FIGURE 1:**
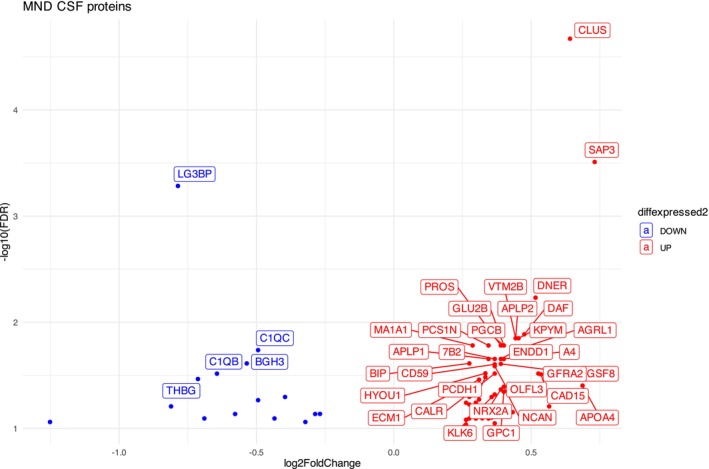
Volcano plot providing an overview of the most significantly differentially expressed ALS CSF proteins. All plotted proteins are significant at the threshold FDR < 0.1. The proteins with FDR < 0.05 are labelled.

**FIGURE 2:**
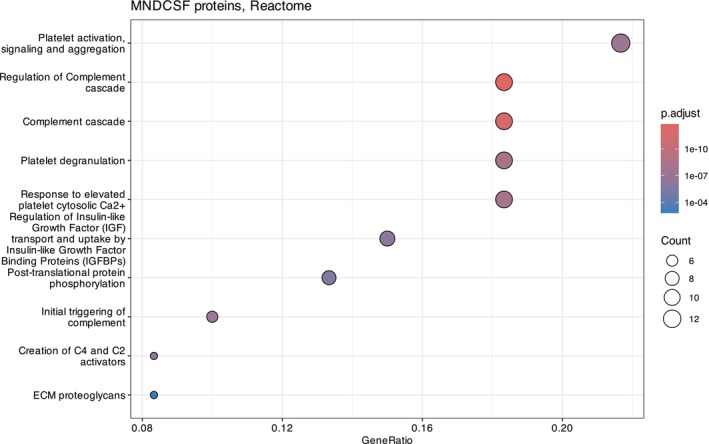
Dot plot visualisation of enrichment terms depicting ALS CSF proteins with enrichment scores (with FDR‐adjusted *p*‐values) and gene ratio. The size of the dots indicates the number of proteins that match the respective Reactome pathway.

**FIGURE 3:**
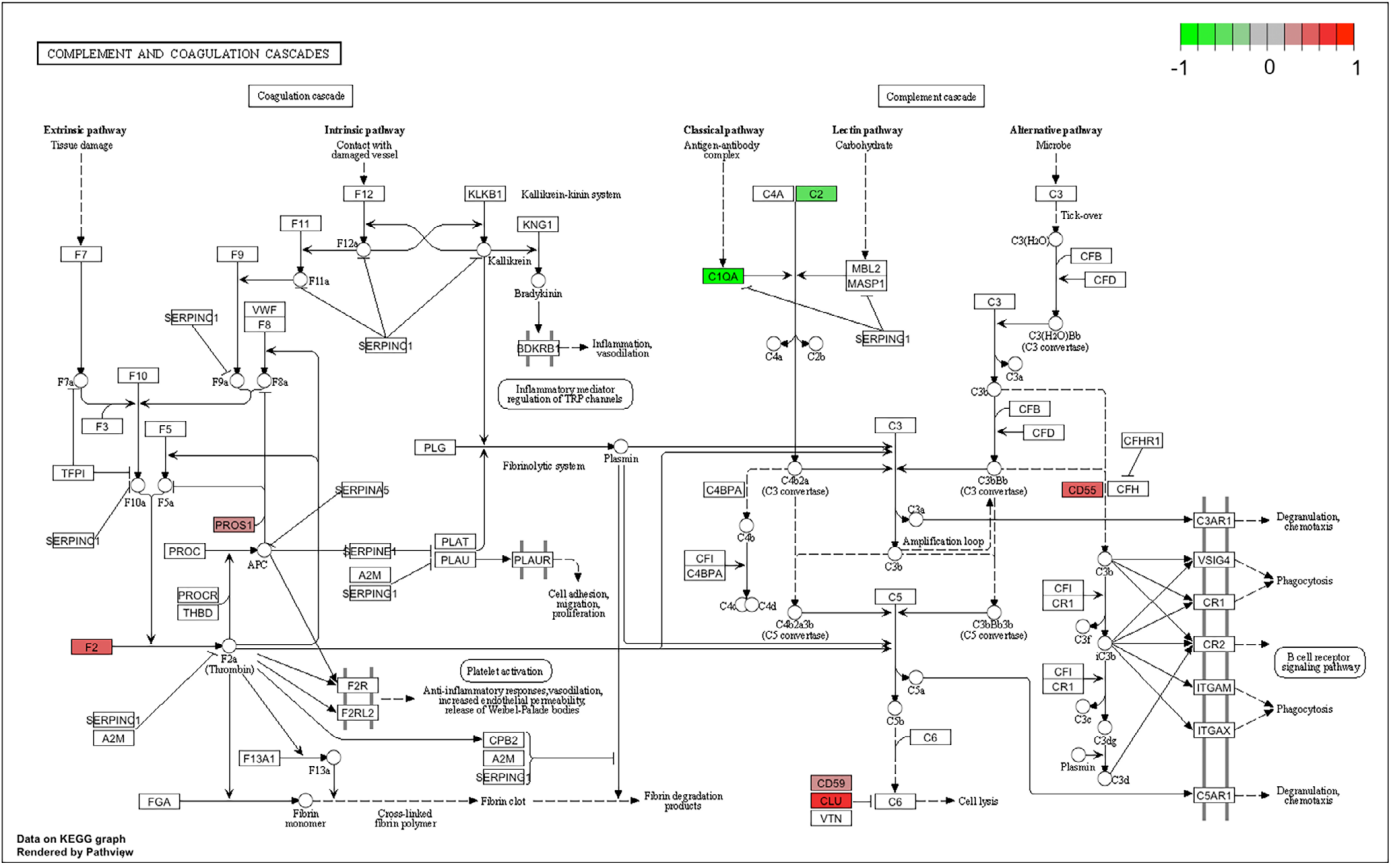
KEGG pathway analysis showing the significantly regulated proteins in the complement and coagulation cascade. The upregulated proteins are shown in red, and the downregulated proteins are in green.

**FIGURE 4:**
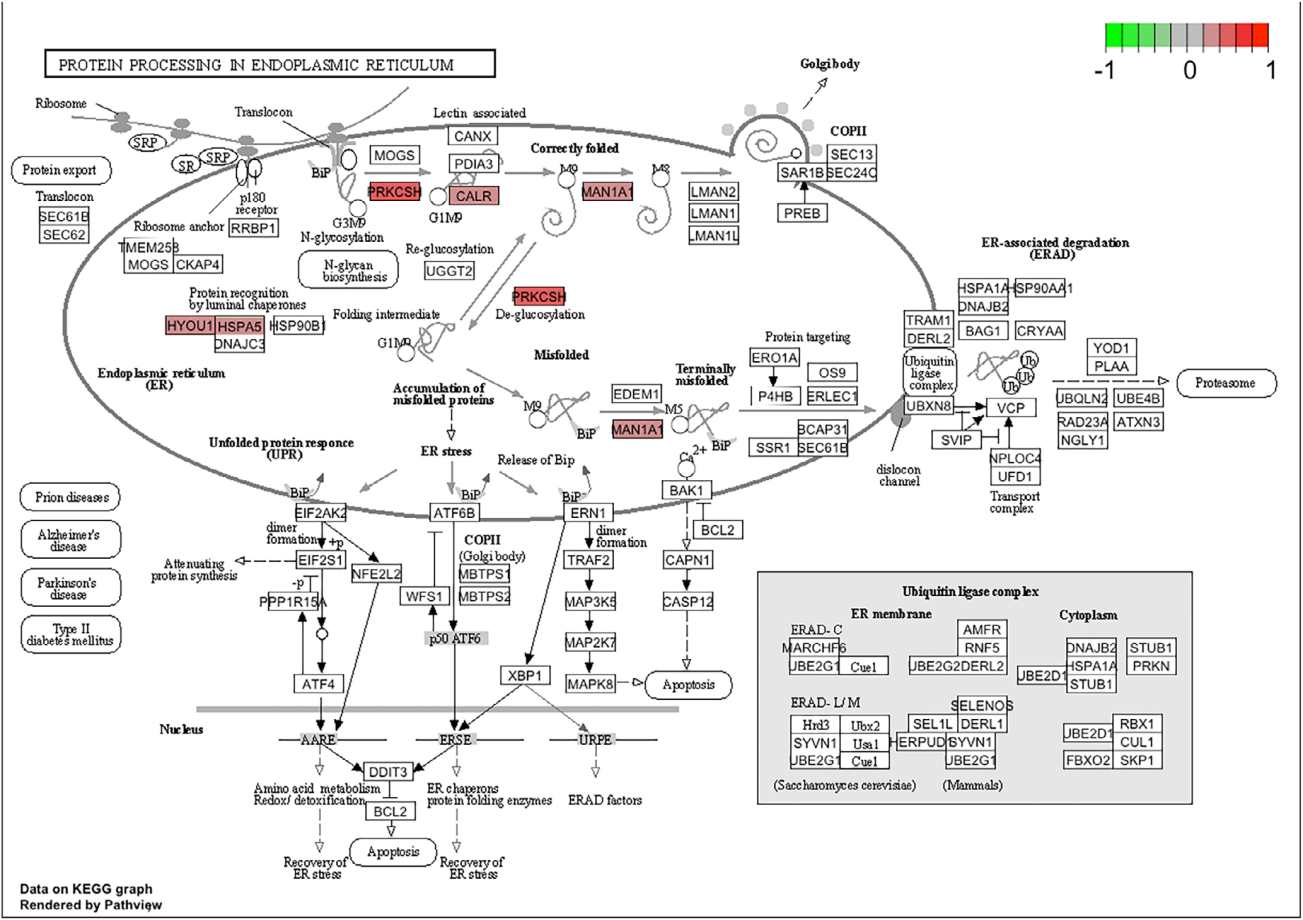
KEGG pathway analysis showing the significantly regulated proteins in the protein processing in the endoplasmic reticulum pathway. The upregulated proteins are shown in red, and the downregulated proteins are in green.

**FIGURE 5:**
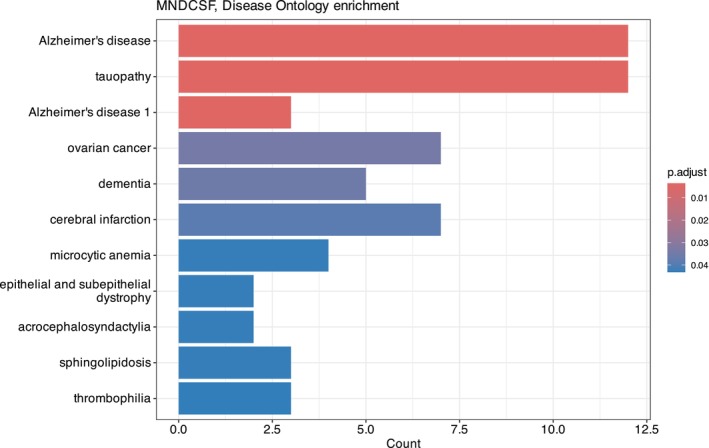
Disease ontology analysis identified the neurological diseases that were significantly affected. Adjusted *p*‐values (FDR) and gene counts are indicated.

ReactomePA identified 47 significantly enriched pathways (FDR < 0.05) (Table 2, Figures 1 and 2), with the most prominent signals involving regulation of the complement cascade and protein processing in the endoplasmic reticulum (ER). Similarly, KEGG analysis identified 11 significantly enriched pathways (FDR < 0.05) (Table 3, Figures 3 and 4), with Complement and Coagulation Cascades emerging as the top‐ranked pathway. DOSE analysis further revealed enrichment of 55 (threshold FDR < 0.05) different diseases (Table 4, Figure 5) and disease‐associated gene sets, including multiple neurodegenerative and protein aggregation‐related disorders. As expected, we observed significant upregulation of APP, including its forms APP beta, amyloid‐like protein 2 and amyloid‐like protein 1.

**TABLE 2:** The enrichment of 49 Reactome pathways in the proteome of the CSF of ALS patients.

ID	Description	GeneRatio	BgRatio	*p*	*p*‐adjust
R‐HSA‐977606	Regulation of Complement cascade	11/60	47/11230	5.78E‐16	1.79E‐13
R‐HSA‐166658	Complement cascade	11/60	58/11230	7.23E‐15	1.12E‐12
R‐HSA‐114608	Platelet degranulation	11/60	129/11230	6.35E‐11	6.54E‐09
R‐HSA‐76005	Response to elevated platelet cytosolic Ca^2+^	11/60	134/11230	9.61E‐11	7.43E‐09
R‐HSA‐76002	Platelet activation, signalling and aggregation	13/60	262/11230	8.77E‐10	5.42E‐08
R‐HSA‐166663	Initial triggering of complement	6/60	23/11230	1.69E‐09	8.72E‐08
R‐HSA‐166786	Creation of C4 and C2 activators	5/60	14/11230	7.09E‐09	3.13E‐07
R‐HSA‐381426	Regulation of Insulin‐like Growth Factor (IGF) transport and uptake by Insulin‐like Growth Factor Binding Proteins (IGFBPs)	9/60	125/11230	1.80E‐08	6.95E‐07
R‐HSA‐8957275	Post‐translational protein phosphorylation	8/60	108/11230	9.52E‐08	3.27E‐06
R‐HSA‐3000178	ECM proteoglycans	5/60	76/11230	5.08E‐05	0.00156875
R‐HSA‐3560782	Diseases associated with glycosaminoglycan metabolism	4/60	41/11230	6.43E‐05	0.00180685
R‐HSA‐3560783	Defective B4GALT7 causes EDS, progeroid type	3/60	20/11230	0.00015495	0.00341994
R‐HSA‐3560801	Defective B3GAT3 causes JDSSDHD	3/60	20/11230	0.00015495	0.00341994
R‐HSA‐4420332	Defective B3GALT6 causes EDSP2 and SEMDJL1	3/60	20/11230	0.00015495	0.00341994
R‐HSA‐977606	Regulation of Complement cascade	11/60	47/11230	5.78E‐16	1.79E‐13
R‐HSA‐9839923	Dengue Virus Infection	9/60	401/11230	0.00025362	0.00518878
R‐HSA‐1474244	Extracellular matrix organisation	8/60	318/11230	0.00026867	0.00518878
R‐HSA‐1971475	Glycosaminoglycan‐protein linkage region biosynthesis	3/60	29/11230	0.00047995	0.00872385
R‐HSA‐9920588	Dengue virus activates/modulates innate and adaptive immune responses	3/60	32/11230	0.00064412	0.01105738
R‐HSA‐6798695	Neutrophil degranulation	9/60	469/11230	0.0007934	0.01290315
R‐HSA‐3781865	Diseases of glycosylation	5/60	145/11230	0.00103377	0.01518949
R‐HSA‐159740	Gamma‐carboxylation of protein precursors	2/60	10/11230	0.00122892	0.01518949
R‐HSA‐159782	Removal of aminoterminal propeptides from gamma‐carboxylated proteins	2/60	10/11230	0.00122892	0.01518949
R‐HSA‐381183	ATF6 (ATF6‐alpha) activates chaperone genes	2/60	10/11230	0.00122892	0.01518949
R‐HSA‐8963888	Chylomicron assembly	2/60	10/11230	0.00122892	0.01518949
R‐HSA‐8963901	Chylomicron remodelling	2/60	10/11230	0.00122892	0.01518949
R‐HSA‐159854	Gamma‐carboxylation, transport, and amino‐terminal cleavage of proteins	2/60	11/11230	0.00149686	0.0177896
R‐HSA‐975634	Retinoid metabolism and transport	3/60	44/11230	0.00164343	0.01784292
R‐HSA‐9918485	Dengue Virus Attachment and Entry	3/60	44/11230	0.00164343	0.01784292
R‐HSA‐418990	Adherens junctions interactions	6/60	240/11230	0.00170831	0.01784292
R‐HSA‐2022923	DS‐GAG biosynthesis	2/60	12/11230	0.00179007	0.01784292
R‐HSA‐381033	ATF6 (ATF6‐alpha) activates chaperones	2/60	12/11230	0.00179007	0.01784292
R‐HSA‐2173782	Binding and Uptake of Ligands by Scavenger Receptors	3/60	46/11230	0.00186946	0.01805198
R‐HSA‐879415	Advanced glycosylation end product receptor signalling	2/60	13/11230	0.00210827	0.01921249
R‐HSA‐6806667	Metabolism of fat‐soluble vitamins	3/60	48/11230	0.002114	0.01921249
R‐HSA‐9918432	Maturation of DENV proteins	3/60	51/11230	0.00251661	0.02221809
R‐HSA‐977225	Amyloid fibre formation	4/60	109/11230	0.00269743	0.0231529
R‐HSA‐421270	Cell–cell junction organisation	6/60	272/11230	0.00319758	0.02670408
R‐HSA‐373760	L1CAM interactions	4/60	120/11230	0.00381348	0.0310096
R‐HSA‐3000480	Scavenging by Class A Receptors	2/60	19/11230	0.00452777	0.03456517
R‐HSA‐8963898	Plasma lipoprotein assembly	2/60	19/11230	0.00452777	0.03456517
R‐HSA‐375165	NCAM signalling for neurite out‐growth	3/60	63/11230	0.00458632	0.03456517
R‐HSA‐446728	Cell junction organisation	6/60	298/11230	0.00499403	0.03564334
R‐HSA‐2024101	CS/DS degradation	2/60	20/11230	0.00501364	0.03564334
R‐HSA‐71387	Metabolism of carbohydrates and carbohydrate derivatives	6/60	299/11230	0.00507543	0.03564334
R‐HSA‐2022870	CS‐GAG biosynthesis	2/60	21/11230	0.00552243	0.0379207
R‐HSA‐1630316	Glycosaminoglycan metabolism	4/60	136/11230	0.00594197	0.03980119
R‐HSA‐140875	Common Pathway of Fibrin Clot Formation	2/60	22/11230	0.0060539	0.03980119
R‐HSA‐140837	Intrinsic Pathway of Fibrin Clot Formation	2/60	23/11230	0.0066078	0.04253771
R‐HSA‐174824	Plasma lipoprotein assembly, remodelling, and clearance	3/60	75/11230	0.00745397	0.04700566

*Note:* FDR‐corrected *p*‐values are shown as ‘*p*‐adjust’, 49 pathways were statistically significantly enriched.

**TABLE 3:** The enrichment of KEGG pathways in the proteome of the CSF of ALS patients identified 13 enriched KEGG pathways.

ID	Description	GeneRatio	BgRatio	*p*	*p*‐adjust
hsa04610	Complement and coagulation cascades	11/41	88/9561	5.32E‐14	4.84E‐12
hsa05133	Pertussis	6/41	78/9561	8.69E‐07	3.95E‐05
hsa05150	*Staphylococcus aureus* infection	6/41	102/9561	4.22E‐06	0.00012816
hsa05322	Systemic lupus erythematosus	6/41	144/9561	3.06E‐05	0.00069676
hsa05171	Coronavirus disease—COVID‐19	7/41	238/9561	5.92E‐05	0.00107736
hsa04148	Efferocytosis	5/41	157/9561	0.00052087	0.00738256
hsa04514	Cell adhesion molecule (CAM) interaction	5/41	160/9561	0.00056789	0.00738256
hsa04141	Protein processing in endoplasmic reticulum	5/41	174/9561	0.00083088	0.00945127
hsa05142	Chagas disease	4/41	103/9561	0.00094718	0.00957703
hsa04979	Cholesterol metabolism	3/41	52/9561	0.00139817	0.01272335
hsa03273	Virion—Lassa virus and SFTS virus	2/41	17/9561	0.00234267	0.01938028
hsa04936	Alcoholic liver disease	4/41	144/9561	0.00324321	0.02392505
hsa05230	Central carbon metabolism in cancer	3/41	71/9561	0.00341786	0.02392505

*Note:* FDR‐corrected *p*‐values are shown as ‘*p*‐adjust’.

**TABLE 4:** The enrichment of Disease Ontology in the proteome of the CSF of ALS patients showed enrichment for 11 diseases.

ID	Description	GeneRatio	BgRatio	*p*	*p*‐adjust
DOID:10652	Alzheimer's disease	12/54	423/8168	1.50E‐05	0.00361452
DOID:680	Tauopathy	12/54	432/8168	1.85E‐05	0.00361452
DOID:0080348	Alzheimer's disease 1	3/54	9/8168	2.23E‐05	0.00361452
DOID:2394	Ovarian cancer	7/54	196/8168	0.00028037	0.0340645
DOID:1307	Dementia	5/54	94/8168	0.00036747	0.0357177
DOID:3526	Cerebral infarction	7/54	214/8168	0.00047774	0.0386971
DOID:11252	Microcytic anaemia	4/54	62/8168	0.0007166	0.04295508
DOID:0060440	Epithelial and subepithelial dystrophy	2/54	2 289 524	0.00088202	0.04295508
DOID:12960	Acrocephalosyndactylia	2/54	2 289 524	0.00088202	0.04295508
DOID:1927	Sphingolipidosis	3/54	29/8168	0.00088385	0.04295508
DOID:2452	Thrombophilia	3/54	30/8168	0.00097748	0.04318688

*Note:* FDR‐corrected *p*‐values are shown as ‘*p*‐adjust’.

At the protein level, pathway enrichment was driven by coordinated alterations in complement system components (including C1q subunits (A, B and C), C1r, C1s, C2 and C4a) and complement regulatory proteins such as CD59, alongside changes in coagulation‐associated proteins. In parallel, ER stress‐related chaperones and protein folding regulators, including BiP and calreticulin, were significantly enriched, consistent with activation of unfolded protein response pathways. We consistently observed elevated levels of Apolipoprotein A (APOA4), which is enriched in pathways such as complement and coagulation cascades.

Additional pathway signals included enrichment of extracellular matrix organisation and cell adhesion processes, driven by differential abundance of cadherin and protocadherin family members as well as adhesion‐associated proteins.

Among the most upregulated proteins observed in the ALS group, we found the Hyaluronan binding protein 2 (HABP2), an extracellular serine protease, also called FSAP (factor VII activating protease), which ranked sixth in the 20% missing values analysis (Table S1, Figure S1). Together with increased Prothrombin levels, these findings point towards broader dysregulation of coagulation–fibrinolysis and vascular–matrix interaction pathways in ALS CSF (Pinto et al. 2019).

Additional support for altered neuronal signalling and repair mechanisms was provided by increased expression of GFRA2 and DNER. GFRA2 mediates glial‐derived neurotrophic factor (GDNF) signalling and contributes to motor neuron survival and maintenance of neuromuscular junctions (Demireva et al. 2011; Suzuki et al. 2008), while DNER functions as a NOTCH1 pathway activator and has been implicated in neurodegenerative disorders including atypical parkinsonian syndromes (Suzuki et al. 2008; Jabbari et al. 2019; Ochneva et al. 2022).

Ranked seventh in the 20% missing values analysis (Table S1, Figure S1), we found the cell adhesion protein Cadherin‐15 (CAD15). Other upregulated cell adhesion proteins included Cadherin‐13 (CAD13), Protocadherin‐1 (PCDH1) and Cadherin‐2 (CADH2), which ranked thirty‐seventh, forty‐fifth and sixty‐second, respectively (Table S1, Figure S1).

Additionally, ReactomePA identified ‘ATF6 Activates Chaperones’ and KEGG revealed ‘Protein Processing in the ER’ as significantly enriched pathways. KEGG analysis also identified pathways associated with diseases not directly related to neurodegeneration, including pertussis, *Staphylococcus aureus* infection, systemic lupus erythematosus, Coronavirus disease—COVID‐19, alcoholic liver disease and Chagas disease.

### 3.3 Statistical Workflow and Curation

Differentially expressed protein list based on (fold changes and FDR) is mapped on three different databases, Reactome, KEGG and Disease Ontology. The outcomes are the pathways that are represented in our original differentially expressed protein list.

The background protein list is a manually curated list of proteins and pathways they are functionally connected to. We used three different collections, one of which is Reactome, which contains 2848 experimentally confirmed and curated pathways. KEGG is another similar collection of pathways that shows protein interactions and includes 314 human‐specific pathways. Finally, we used the Disease Ontology database, which comprises 11 000 disease terms. Our protein list is the list of proteins that were statistically significantly changed in our study.

Reactome enrichment analysis determines whether genes identified in an experiment are statistically over‐represented in known biological pathways. In a typical workflow, genes of interest, such as differentially expressed genes from transcriptomic analysis, are first mapped to standard identifiers and compared with an appropriate background set, usually all genes detected or tested in the experiment. Each Reactome pathway is then tested to assess whether it contains more genes from the input list than expected by chance, most commonly using a hypergeometric or Fisher's exact test. Because many pathways are tested simultaneously, the resulting *p*‐values are corrected for multiple comparisons, using the Benjamini–Hochberg false discovery rate. Significantly enriched pathways are then ranked and interpreted according to their adjusted *p*‐values, gene ratios, pathway size and the identity of the overlapping genes. This analysis provides a pathway‐level interpretation of gene expression changes, helping to identify biological processes, signalling cascades, and disease‐relevant mechanisms that may be altered in the studied condition.

KEGG pathway enrichment analysis is used to identify biological pathways that are statistically over‐represented among a set of genes of interest, such as differentially expressed genes from transcriptomic analysis. In this workflow, the input genes are first converted to standard identifiers, commonly Entrez Gene IDs, and then compared with curated KEGG pathway annotations. For each KEGG pathway, the analysis tests whether the number of genes from the input list found in that pathway is greater than expected by chance, usually using a hypergeometric or Fisher's exact test. The background set should represent all genes that were detectable or tested in the experiment, rather than the whole genome, to avoid biased results. Because many pathways are tested simultaneously, raw *p*‐values are adjusted for multiple testing, commonly using the Benjamini–Hochberg false discovery rate. Significantly enriched KEGG pathways provide a pathway‐level interpretation of the gene list and help identify altered biological processes, signalling networks, metabolic pathways and disease‐associated mechanisms relevant to the experimental condition.

Disease Ontology enrichment analysis using DOSE is used to determine whether a set of genes of interest, such as differentially expressed genes from transcriptomic analysis, is statistically associated with specific disease categories or phenotypes. In this workflow, genes are first mapped to standard identifiers, usually Entrez Gene IDs, and then compared with curated gene–disease annotations from the Disease Ontology database. For each disease term, DOSE tests whether the number of input genes annotated to that disease is greater than expected by chance, commonly using a hypergeometric statistical test. The analysis requires an appropriate background gene universe, ideally all genes detected or tested in the experiment, to ensure that the enrichment results are not biased. Because many disease terms are tested simultaneously, raw *p*‐values are corrected for multiple testing, using the Benjamini–Hochberg false discovery rate. Significantly enriched disease ontology terms provide a disease‐level interpretation of the gene list and help identify pathological processes, disease associations and clinically relevant mechanisms that may be linked to the molecular changes observed in the study.

## 4 Discussion

### 4.1 Overview and Study Context

Amyotrophic lateral sclerosis (ALS) is increasingly recognised as a multisystem disorder in which degeneration of the motor unit arises from dysfunction across interacting neuronal, glial, vascular and muscular compartments (McCann et al. 2020). Although motor neuron loss remains the defining pathological feature, growing evidence suggests that disease progression reflects a broader breakdown of the microenvironments that normally maintain neuromuscular connectivity and function.

Cerebrospinal fluid (CSF) proteomics provides a valuable window into molecular processes occurring at the interface of the central nervous system, interstitial fluid dynamics and vascular exchange. This is particularly relevant in ALS, where motor neuron vulnerability is closely linked to high metabolic demand, blood–spinal cord barrier integrity and adequate vascular support of long corticospinal axons and neuromuscular terminals. Nevertheless, interpretation of CSF proteomic signatures remains challenging because of biological heterogeneity among patient cohorts and differences in analytical depth across studies.

Neurologically diseased controls were included in the present study to reflect the clinical reality of ALS diagnosis, which occurs within a spectrum of neurological disorders rather than against a healthy physiological background. Although this approach introduces additional biological variability, it enhances the clinical relevance of the identified pathways and strengthens the translational value of the findings.

Recent CSF proteomic studies, including Trautwig et al. (2025), have consistently identified immune activation, proteostasis impairment and synaptic dysfunction as core features of ALS pathology. The present findings build upon this framework by highlighting a coordinated neurovascular–synaptic axis that links vascular regulation, extracellular matrix remodelling and maintenance of the motor unit. Collectively, these observations support a broader systems‐level view of ALS pathogenesis in which vascular, inflammatory and synaptic processes interact to drive disease progression.

### 4.2 Mechanistic Hypothesis: Neurovascular Unit Failure as a Driver of Motor Unit Degeneration

A central mechanistic interpretation emerging from these data is that ALS involves progressive dysfunction of the neurovascular unit, resulting in impaired metabolic support, compromised barrier integrity and secondary destabilisation of motor neurons and neuromuscular junctions.

Within this framework, early neurovascular dysfunction may initiate a feed‐forward cycle of degeneration. Vascular and endothelial dysregulation can impair nutrient and oxygen delivery to motor structures, while disruption of blood–spinal cord barrier integrity may promote chronic innate immune activation. Concurrent inflammatory and coagulation‐related signalling may drive extracellular matrix remodelling, ultimately destabilising synaptic and axonal compartments through loss of trophic and metabolic support.

This model suggests that motor neuron degeneration is not solely a cell‐autonomous process. Rather, it emerges from progressive failure of vascular–glial–neuronal coupling within the spinal cord and motor cortex microenvironment. Consequently, dysfunction of the neurovascular unit may represent an important upstream contributor to motor unit degeneration.

### 4.3 Neuroinflammation and Vascular‐Linked Immune Dysregulation

The immune alterations observed in ALS CSF are most appropriately interpreted not simply as neuroinflammation, but as evidence of vascular‐associated innate immune activation. Complement pathway dysregulation suggests sustained activation of humoral innate immunity, a process closely linked to endothelial signalling, vascular injury responses and disruption of blood–CNS barrier integrity. These findings are consistent with previous ALS proteomic studies identifying complement activation as a hallmark of disease‐associated neuroinflammation (Trautwig et al. 2025; Luo et al. 2025; Doss et al. 2022).

Importantly, the observed pattern does not indicate a simple increase in inflammatory activity. Rather, it is consistent with impaired resolution of immune–vascular signalling, whereby regulatory mechanisms fail to restore homeostasis. Such dysfunction may contribute to persistent microglial activation and endothelial stress signalling, thereby reinforcing a chronic inflammatory vascular niche.

From a mechanistic perspective, complement activation at the neurovascular interface may contribute directly to disease progression through several pathways. These include promotion of endothelial dysfunction and increased barrier permeability, opsonisation and pruning of synaptic structures—including neuromuscular junctions—and recruitment of peripheral immune mediators into the CNS compartment.

Additional inflammatory signatures further support this interpretation (Calma et al. 2024). Reduced levels of the scavenger receptor CD163 were observed in ALS CSF, consistent with findings by Mamoor (2022), who reported significantly decreased CD163 expression in both motor neurons and skeletal muscle from ALS patients. Given the role of CD163 in macrophage‐mediated inflammatory regulation, reduced expression may contribute to sustained or inadequately resolved neuroinflammatory activity.

Similarly, decreased Galectin‐3‐binding protein (Gal‐3BP), an important regulator of immune signalling and cell–cell interactions, may impair tolerance to self‐antigens and exacerbate neurodegenerative processes (Doss et al. 2022; Mambetsariev et al. 2010). In parallel, increased levels of latent transforming growth factor β‐binding protein 4 (LTBP4), which maintains TGFβ in an inactive extracellular matrix‐bound state, suggest further dysregulation of anti‐inflammatory signalling pathways. Altered expression of inflammatory mediators, including Alpha‐1‐antichymotrypsin and Prothrombin, further supports the presence of an imbalanced inflammatory environment.

The observed increase in lipoprotein A, recently associated with ALS (Calma et al. 2024; Meyer 2014), together with elevated expression of Ly‐6/neurotoxin‐like protein 1 (LYNX1), a regulator of synaptic plasticity (Meyer 2014; Mamoor 2022; Loimaranta et al. 2018), suggests that inflammatory and synaptic disturbances may develop concurrently during disease progression.

These findings closely align with observations reported by Trautwig et al. (2025), who likewise identified complement activation and immune dysregulation as dominant ALS‐associated CSF signatures. However, the present study extends these observations by demonstrating coordinated dysregulation across both upstream complement components and regulatory proteins, suggesting a more complex and potentially stage‐dependent inflammatory response.

Taken together, these findings support a model in which immune dysregulation in ALS is tightly linked to vascular barrier dysfunction rather than being restricted to parenchymal inflammation alone.

### 4.4 Extracellular Matrix Remodelling and Neurovascular–Neuromuscular Coupling

A second major mechanistic axis identified in this study involves disruption of the extracellular matrix (ECM) and adhesion systems, which are essential not only for synaptic stability but also for maintaining neurovascular integrity. The observed alterations suggest that structural remodelling occurs across both vascular and neuromuscular compartments, potentially linking neurovascular dysfunction to motor unit degeneration.

The ECM plays a critical role in regulating endothelial stability, perivascular support, diffusion of metabolic substrates, structural anchoring of neuromuscular junctions, and retrograde trophic signalling from muscle to motor neuron. Consequently, disruption of ECM and adhesion networks may compromise both vascular–parenchymal coupling and neuromuscular synaptic maintenance.

Consistent with this interpretation, increased coagulation‐ and protease‐associated signalling suggests activation of a vascular injury response phenotype. These pathways are closely linked to endothelial activation and blood–CNS barrier remodelling and may therefore reflect a chronic imbalance between microvascular injury and repair. Such alterations could impair capillary perfusion within motor regions, modify the extracellular matrix composition of perivascular spaces, and compromise the structural integrity of the synaptic basal lamina at the neuromuscular junction.

Further support for this hypothesis comes from the differential expression of multiple cadherin and protocadherin family members, including CADH2, CADH13, CADH15 and PCDH1. These proteins are central regulators of cell–cell adhesion and tissue organisation, and their altered expression suggests widespread remodelling of structural interactions within the ALS nervous system.

Cadherin‐mediated adhesion is fundamental to spinal cord development and motor neuron organisation (Mambetsariev et al. 2010; Demireva et al. 2011). Experimental studies have shown that disruption of these pathways can result in abnormal motor column segregation and impaired neuronal connectivity. The coordinated upregulation of cadherin‐family proteins observed in our dataset therefore suggests ongoing adhesion‐related signalling associated with disease‐driven neuronal remodelling and degeneration.

Collectively, these findings provide a direct mechanistic link between vascular dysfunction and motor unit failure. Although previous ALS proteomic studies, including Trautwig et al. (2025), have reported alterations in cell adhesion and extracellular signalling pathways, our data additionally reveal a coordinated cadherin‐family and coagulation‐associated signature that may represent a previously underappreciated component of ALS‐associated neurovascular and ECM dysregulation.

### 4.5 Endoplasmic Reticulum Stress, Metabolic Vulnerability and Neurovascular Coupling

Endoplasmic reticulum (ER) stress and proteostasis impairment can be integrated into the proposed neurovascular model through disruption of metabolic coupling. Motor neurons are uniquely dependent on continuous vascular‐derived metabolic support because of their large size, extensive axonal projections, and high synaptic energy requirements.

Within this framework, impaired neurovascular support may result in chronic energetic stress, reduced ATP availability, impaired protein folding capacity, activation of unfolded protein response pathways, and subsequent synaptic and axonal dysfunction. ER stress may therefore represent not only an intracellular pathological process but also a downstream consequence of vascular and metabolic insufficiency within the motor unit niche. Furthermore, proteostasis failure may extend beyond the cell through altered secretion and turnover of synaptic and ECM proteins, linking intracellular stress responses to broader neurovascular and neuromuscular instability.

Consistent with this interpretation, ALS CSF demonstrated increased abundance of key ER chaperones, including BiP (HSPA5) and calreticulin, both of which play central roles in adaptive responses to misfolded protein accumulation (Ochneva et al. 2022; Francois‐Moutal et al. 2022; Thomson and Williams 2005; Zinkie et al. 2013). Elevated expression of these chaperones is consistent with activation of ER stress pathways and ongoing attempts to restore protein homeostasis (Francois‐Moutal et al. 2022; Gomez‐Almeria et al. 2021).

BiP is of particular relevance to ALS because it directly interacts with TDP‐43, the aggregation‐prone RNA‐binding protein that accumulates pathologically in affected neurons (Gomez‐Almeria et al. 2021). Experimental evidence demonstrates that partial deletion of BiP accelerates neurological decline and reduces survival in ALS mouse models, suggesting that increased BiP expression may represent an endogenous protective response to proteotoxic stress (Bracher and Verghese 2015).

In parallel, increased expression of APP and the related amyloid precursor‐like proteins APLP1 and APLP2 further supports the contribution of protein misfolding and aggregation to ALS pathology. Similar pathogenic mechanisms are well established in Alzheimer's disease (Thomson and Williams 2005). Moreover, Bryson et al. (2012) demonstrated that APP upregulation coincides with symptom onset in ALS models and contributes to β‐amyloid accumulation within motor neurons and glial cells (Zinkie et al. 2013; Thapa et al. 2007).

Several additional proteins associated with ER stress and synaptic proteostasis, including PCS1N, SCG1 and 7B2, were also increased in ALS CSF. The involvement of APLP1 and APLP2 in neuronal homeostasis and synaptic function further reinforces the close relationship between dysregulated protein processing and synaptic dysfunction in ALS (Thompson et al. 2020).

These observations closely mirror the findings of Trautwig et al. (2025), who identified proteostasis imbalance and ER stress as central features of ALS CSF pathology. However, our data additionally suggest coordinated activation of extracellular protein clearance pathways occurring alongside intracellular stress responses, indicating a broader and more integrated proteostatic disturbance.

### 4.6 Neuroprotective and Neurovascular–Synaptic Compensatory Responses

Alongside pathways associated with degeneration, the present data also suggest activation of compensatory mechanisms aimed at preserving neurovascular and neuromuscular homeostasis. These responses include extracellular chaperone activity, synaptic repair signalling and neurotrophic support pathways.

Mechanistically, such responses may reflect attempts to stabilise synaptic protein environments during inflammatory stress, reinforce neuromuscular junction structure in the context of ECM disruption, and activate regenerative signalling within compromised neurovascular niches. Nevertheless, the persistence of these pathways likely indicates an ultimately unsuccessful repair response, in which compensatory mechanisms are unable to counteract ongoing structural and metabolic deterioration.

Several proteins upregulated in ALS CSF appear consistent with these endogenous protective responses. Among them, Clusterin was significantly increased. Clusterin is a secreted extracellular chaperone expressed throughout the nervous system that inhibits aggregation of non‐native proteins and suppresses amyloid fibril formation (Thompson et al. 2020; Mohanty et al. 2020). While reduced Clusterin levels have previously been associated with ALS pathology, increased CSF concentrations have been proposed as a marker of disease activity (Klíčová et al. 2024; Mitchell et al. 2020). The elevated levels observed in our cohort may therefore represent an adaptive response to extracellular proteotoxic stress and APP accumulation.

Similarly, Secretogranin‐2 (SCG2), a precursor of the neuroactive peptide secretoneurin, was increased in ALS CSF. Secretoneurin promotes neurite outgrowth and contributes to neuronal repair and plasticity (Shyu et al. 2008; Hsieh et al. 2022), suggesting activation of regenerative pathways within the diseased nervous system. Increased GM2A expression may likewise reflect adaptive responses, although excessive GM2A activity has been associated with reduced neurite integrity and neuronal dysfunction in neurodegenerative disorders (Wang et al. 2017).

Taken together, these findings indicate that ALS CSF contains molecular signatures not only of neurodegeneration and inflammation, but also of endogenous protective mechanisms attempting to counteract neuronal injury and proteotoxic stress.

### 4.7 Integrated Mechanistic Model and Study Implications in Relation to ALS Proteomics Literature and Study Novelty

Collectively, the present findings support a unified model of ALS as a disorder of neurovascular–neuromuscular unit failure in which vascular dysfunction acts as an upstream or parallel driver of motor system degeneration.

Within this model, neurovascular instability initiates or amplifies immune activation, while immune–vascular crosstalk promotes extracellular matrix remodelling and synaptic destabilisation. Progressive metabolic insufficiency then triggers ER stress and proteostasis collapse, ultimately contributing to neuromuscular junction failure through the combined effects of structural disruption, chronic inflammation and energetic stress. This framework shifts emphasis away from a purely neuron‐centric view of ALS towards a systems‐level model of vascular‐supported motor unit failure that integrates both CNS and peripheral neuromuscular pathology.

Overall, our findings are highly consistent with the growing ALS CSF proteomics literature identifying complement activation, immune dysregulation and impaired proteostasis as central disease‐associated processes (Raghunathan et al. 2022; Trautwig et al. 2025; Luo et al. 2025). In particular, the strong enrichment of complement pathways and ER stress‐related proteins closely parallels the molecular signatures recently reported by Trautwig et al. (2025).

At the same time, the present study provides several additional insights. First, we demonstrate integrated dysregulation across complement, coagulation, extracellular matrix, and ER stress pathways within a single CSF cohort, supporting the concept of ALS as a systems‐level disorder involving interacting inflammatory, structural and proteostatic mechanisms. Second, we provide more detailed resolution of complement dysregulation through the identification of coordinated alterations in both activating and inhibitory components of the cascade. Third, we identify less frequently reported candidate proteins and pathway clusters, including coordinated cadherin‐family upregulation and HABP2‐associated coagulation–fibrinolysis signalling, suggesting broader neurovascular and extracellular matrix involvement than previously appreciated.

Comparison with previously published datasets confirms complement activation, inflammatory signalling and cell adhesion dysregulation as recurrent molecular features of ALS. Nevertheless, the partial overlap between studies highlights the biological complexity and heterogeneity of ALS and underscores the ongoing challenge of identifying universally reproducible proteomic biomarkers (Raghunathan et al. 2022; Trautwig et al. 2025).

Interestingly, KEGG analysis also identified pathways associated with infectious and systemic inflammatory diseases, including pertussis, *S. aureus* infection, Coronavirus disease and Chagas disease. These findings most likely reflect activation of shared inflammatory and complement‐related pathways rather than disease‐specific infectious mechanisms.

## 5 Limitations

Several limitations should be considered when interpreting these findings. First, the relatively small sample size limits statistical power to detect subtle biological changes. In addition, the use of neurologically diseased controls introduces heterogeneity that may obscure weaker ALS‐specific signals. However, this design also enhances clinical relevance and may improve the translational value of identified biomarkers and pathways.

A further limitation relates to the intrinsic characteristics of CSF proteomics. CSF underrepresents many low‐abundance vascular and synaptic regulatory proteins, particularly those involved in dynamic endothelial signalling and neuromuscular junction maintenance. Consequently, important aspects of neurovascular dysfunction may remain incompletely captured. Future studies employing more sensitive technologies and spatially resolved approaches, including vascular‐enriched proteomics and single‐cell‐resolved vascular transcriptomics, will be required to more fully characterise neurovascular contributions to ALS pathology. Integration of CSF proteomics with vascular biomarkers and peripheral nerve–muscle omics will also be essential for validating the proposed neurovascular–neuromuscular model.

An additional limitation concerns proteome depth. The relatively modest coverage achieved in this study (1223 proteins identified in total and 186–348 proteins quantitatively analysed depending on missingness thresholds) reflects both biological and technical constraints inherent to CSF proteomics. CSF protein concentrations are several orders of magnitude lower than those observed in plasma, substantially limiting detectable peptide complexity.

Although the Orbitrap Q Exactive Plus platform used in this study is robust and widely validated for quantitative proteomics, it lacks the sensitivity and ion utilisation efficiency of newer‐generation instruments such as Orbitrap Eclipse, Exploris and Astral systems, as well as trapped ion mobility‐enabled platforms including timsTOF SCP. As a result, low‐abundance CSF proteins may not have been fully captured.

Compared with recent deep‐fractionated and advanced data‐independent acquisition (DIA) workflows, which frequently report identification of approximately 2000–5000 CSF proteins depending on analytical depth, the proteome coverage achieved here is comparatively lower. For example Schutzer et al. (2010) reported approximately 2600 proteins in normal human CSF using extensive fractionation approaches. In contrast, single‐shot or moderate‐depth LC–MS/MS studies typically quantify approximately 500–1500 proteins, placing the present study within the expected range for non‐fractionated discovery proteomics workflows.

Further validation in independent cohorts and through complementary targeted or affinity‐based approaches will be necessary to confirm the most promising biomarker candidates and determine their diagnostic and prognostic utility.

Overall, these findings highlight the complex and interconnected biological landscape of ALS CSF and further support the value of proteomic profiling for identifying disease‐associated molecular pathways and potential therapeutic targets.

## Author Contributions

**Pille Taba:** conceptualization, investigation, funding acquisition, writing – review and editing, methodology, validation, project administration, supervision, resources. **Karin Rallmann:** conceptualization, investigation, writing – review and editing, methodology, formal analysis. **Eleonora Sabetta:** writing – original draft, validation, visualization, writing – review and editing, data curation. **Bal Hari Poudel:** writing – review and editing, investigation. **Massimo Locatelli:** investigation, writing – review and editing. **Sulev Kõks:** investigation, funding acquisition, writing – review and editing, formal analysis, project administration, supervision, resources. **Abigail L. Pfaff:** writing – review and editing, investigation. **Jonas Bergquist:** conceptualization, investigation, funding acquisition, writing – review and editing, methodology, validation, formal analysis, project administration, supervision, resources. **Davide Ferrari:** investigation, writing – review and editing.

## Funding

This research was funded by MSWA, the Perron Institute for Neurological and Translational Science, the Estonian Research Council Grants PRG2736 and TEM‐TA110, the SA EUS 100a Fund, and by a donation from an anonymous private donor to Professor Jonas Bergquist.

## Conflicts of Interest

The authors declare no conflicts of interest.

## Supporting information


**Table S1:** List of significantly regulated proteins in the ALS group versus non‐ALS group (20% missing values).
**Table S2:** List of significantly regulated proteins in the ALS group versus non‐ALS group (no missing values).
**Table S3:** (A) Coefficients of variation of the analysed proteins in ALS (20% missing values), (B) Coefficients of variation of the analysed proteins in non‐ALS group (20% missing values), (C) Coefficients of variation of the analysed proteins in ALS (no missing values), (D) Coefficients of variation of the analysed proteins in non‐ALS group (no missing values).
**Figure S1:** Boxplots for significant (*p* < 0.05) differences between patients and controls, allowing for 20% missing values.
**Figure S2:** Boxplots for significant (*p* < 0.05) differences between patients and controls, with no missing values.

## Data Availability

The data presented in this study are available in Supporting Information and on request to the corresponding author.
