# Supplementary material for: Comparison of Proteomic Analysis of Cerebrospinal Fluid From Neurological Patients With and Without Amyotrophic Lateral Sclerosis
Source: J Neurochem. 2026 Jun 26;170(6):e70508. doi: 10.1111/jnc.70508 (PMC13307639; doi:10.1111/jnc.70508)
Supplement: Supplementary file 1 — Table S1: List of significantly regulated proteins in the ALS group versus non‐ALS group (20% missing values). Table S2: List of significantly regulated proteins in the ALS group versus non‐ALS group (no missing values). Table S3: (A) Coefficients of variation of the analysed proteins in ALS (20% missing values), (B) Coefficients of variation of the analysed proteins in non‐ALS group (20% missing values), (C) Coefficients of variation of the analysed proteins in ALS (no missing values), (D) Coefficients of variation of the analysed proteins in non‐ALS group (no missing values). Figure S1: Boxplots for significant (p < 0.05) differences between patients and controls, allowing for 20% missing values. Figure S2: Boxplots for significant (p < 0.05) differences between patients and controls, with no missing values. [file JNC-170-e70508-s001.docx]

**SUPPLEMENTARY INFORMATION FOR THE MANUSCRIPT**

**Comparison of proteomic analysis of cerebrospinal fluid from neurological patients with and without amyotrophic lateral sclerosis**

**Eleonora Sabetta^1^, Karin Rallmann^2^, Pille Taba^2,3^, Abigail L. Pfaff^4,5^, Bal Hari Poudel^5^, Davide Ferrari^6^, Massimo Locatelli^1^, Sulev Kõks^4,5#^, Jonas Bergquist^7, 8^*^#^**

**1. IRCCS Ospedale San Raffaele, Via Olgettina 60, 20132 Milan, Italy;** [**sabetta.eleonora@hsr.it**](mailto:sabetta.eleonora@hsr.it)**, locatelli.massimo@hsr.it**

**2. Department of Neurology, Tartu University Hospital, Tartu, Estonia; karin.Rallmann@kliinikum.ee, pille.taba@ut.ee**

**3. Institute of Clinical Medicine, University Tartu, Tartu, Estonia; pille.taba@ut.ee**

**4. Perron Institute for Neurological and Translational Science, Perth, WA 6009, Australia; abigail.pfaff@uwa.edu.au, sulev.koks@uwa.edu.au**

**5. Centre for Molecular Medicine and Innovative Therapeutics, Murdoch University, Perth, WA 6150, Australia; abigail.pfaff@uwa.edu.au, sulev.koks@uwa.edu.au,** [**bal.poudel@murdoch.edu.au**](mailto:bal.poudel@murdoch.edu.au)

**6. SCVSA Department, University of Parma, Parma, Italy**

**7. Analytical Chemistry and Neurochemistry, Department of Chemistry for Life Sciences, Biomedical Center, Uppsala University, Uppsala Sweden; jonas.bergquist@kemi.uu.se**

**8. The OMF ME/CFS collaborative research center at Uppsala University, Biomedical Center, Uppsala Sweden; jonas.bergquist@kemi.uu.se**

**^#^ Authors share the last author position**

*** Correspondence to:**

**Email:** [**jonas.bergquist@kemi.uu.se**](mailto:jonas.bergquist@kemi.uu.se)

**Tel: +46 18 471 3675**

**Supplementary Table 1.** List of significantly regulated proteins in the ALS group vs non-ALS group (20% missing values).

| **Protein name** | **Uniprot_name** | **Ratio** | **p-value** |
| --- | --- | --- | --- |
| **Upregulated proteins** | | | |
| Ganglioside GM2 activator | SAP3_HUMAN | 1.66 | 1.78E-06 |
| Apolipoprotein A-IV | APOA4_HUMAN | 1.61 | 0.006 |
| Ly-6/neurotoxin-like protein 1 | LYNX1_HUMAN | 1.60 | 0.0004 |
| Clusterin | CLUS_HUMAN | 1.56 | 6.13E-08 |
| Prothrombin | THRB_HUMAN | 1.49 | 0.013 |
| Hyaluronan-binding protein 2 | HABP2_HUMAN | 1.48 | 0.012 |
| Latent-transforming growth factor beta-binding protein 4 | LTBP4_HUMAN | 1.46 | 0.0003 |
| Cadherin-15 | CAD15_HUMAN | 1.45 | 0.005 |
| GDNF family receptor alpha-2 | GFRA2_HUMAN | 1.44 | 0.004 |
| Delta and Notch-like epidermal growth factor-related receptor | DNER_HUMAN | 1.43 | 0.001 |
| Complement decay-accelerating factor | DAF_HUMAN | 1.39 | 0.001 |
| Pyruvate kinase PKM | KPYM_HUMAN | 1.37 | 0.001 |
| V-set and transmembrane domain-containing protein 2B | VTM2B_HUMAN | 1.36 | 0.001 |
| Amyloid-like protein 2 | APLP2_HUMAN | 1.36 | 0.001 |
| Neurosecretory protein VGF | VGF_HUMAN | 1.35 | 0.012 |
| Adhesion G protein-coupled receptor L1 | AGRL1_HUMAN | 1.35 | 0.002 |
| Galectin-1 | LEG1_HUMAN | 1.33 | 0.025 |
| Repulsive guidance molecule A | RGMA_HUMAN | 1.32 | 0.0003 |
| Glypican-1 | GPC1_HUMAN | 1.32 | 0.007 |
| Glucosidase 2 subunit beta | GLU2B_HUMAN | 1.32 | 0.001 |
| Amyloid-beta precursor protein | A4_HUMAN | 1.32 | 0.002 |
| Brevican core protein | PGCB_HUMAN | 1.32 | 0.001 |
| Neurocan core protein | NCAN_HUMAN | 1.32 | 0.006 |
| Vitamin K-dependent protein S | PROS_HUMAN | 1.31 | 0.001 |
| Semaphorin-7A | SEM7A_HUMAN | 1.31 | 0.034 |
| Neurexin-2 | NRX2A_HUMAN | 1.31 | 0.006 |
| Immunoglobulin superfamily member 8 | IGSF8_HUMAN | 1.31 | 0.003 |
| Endonuclease domain-containing 1 protein | ENDD1_HUMAN | 1.31 | 0.002 |
| CD59 glycoprotein | CD59_HUMAN | 1.29 | 0.003 |
| Neuroendocrine protein 7B2 | 7B2_HUMAN | 1.29 | 0.002 |
| Olfactomedin-like protein 3 | OLFL3_HUMAN | 1.29 | 0.003 |
| Kallikrein-6 | KLK6_HUMAN | 1.29 | 0.007 |
| Lymphocyte antigen 6H | LY6H_HUMAN | 1.29 | 0.020 |
| Calreticulin | CALR_HUMAN | 1.29 | 0.004 |
| Oligodendrocyte-myelin glycoprotein | OMGP_HUMAN | 1.28 | 0.015 |
| Protein kinase C-binding protein NELL2 | NELL2_HUMAN | 1.28 | 0.012 |
| Cadherin-13 | CAD13_HUMAN | 1.28 | 0.008 |
| ProSAAS | PCS1N_HUMAN | 1.27 | 0.001 |
| Opioid-binding protein/cell adhesion molecule | OPCM_HUMAN | 1.27 | 0.016 |
| Fibroblast growth factor receptor 2 | FGFR2_HUMAN | 1.27 | 0.0003 |
| Amyloid-like protein 1 | APLP1_HUMAN | 1.27 | 0.002 |
| Apolipoprotein E | APOE_HUMAN | 1.27 | 0.014 |
| Extracellular matrix protein 2 | ECM2_HUMAN | 1.26 | 0.049 |
| Hypoxia up-regulated protein | HYOU1_HUMAN | 1.26 | 0.003 |
| Protocadherin-1 | PCDH1_HUMAN | 1.26 | 0.005 |
| Neuritin | NRN1_HUMAN | 1.25 | 0.026 |
| Fibroblast growth factor receptor 1 | FGFR1_HUMAN | 1.25 | 0.015 |
| Extracellular matrix protein 1 | ECM1_HUMAN | 1.24 | 6.74E-05 |
| Nectin-1 | NECT1_HUMAN | 1.24 | 0.008 |
| Mimecan | MIME_HUMAN | 1.23 | 0.010 |
| Neurofascin | NFASC_HUMAN | 1.23 | 0.016 |
| Secretogranin-2 | SCG2_HUMAN | 1.23 | 0.015 |
| EGF-containing fibulin-like extracellular matrix protein 1 | FBLN3_HUMAN | 1.23 | 0.011 |
| Dystroglycan | DAG1_HUMAN | 1.22 | 0.000 |
| CCN family member 3 | CCN3_HUMAN | 1.22 | 0.031 |
| RGM domain family member B | RGMB_HUMAN | 1.23 | 0.026 |
| DOMON domain-containing protein FRRS1L | FRS1L_HUMAN | 1.22 | 0.049 |
| Tumor necrosis factor receptor superfamily member 21 | TNR21_HUMAN | 1.22 | 0.012 |
| Mannosyl-oligosaccharide 1.2-alpha- mannosidase IA | MA1A1_HUMAN | 1.22 | 0.001 |
| NPC intracellular cholesterol transporter 2 | NPC2_HUMAN | 1.21 | 0.011 |
| Protein FAM3C | FAM3C_HUMAN | 1.21 | 0.017 |
| Cadherin-2 | CADH2_HUMAN | 1.21 | 0.007 |
| Endoplasmic reticulum chaperone BiP | BIP_HUMAN | 1.21 | 0.002 |
| Insulin-like growth factor-binding protein 2 | IBP2_HUMAN | 1.20 | 0.025 |
| Testican-3 | TICN3_HUMAN | 1.20 | 0.010 |
| BDNF/NT-3 growth factors receptor | NTRK2_HUMAN | 1.20 | 0.024 |
| Cell growth regulator with EF hand domain protein 1 | CGRE1_HUMAN | 1.20 | 0.018 |
| Collagen alpha-1(III) chain | CO3A1_HUMAN | 1.19 | 0.038 |
| Dickkopf-related protein 3 | DKK3_HUMAN | 1.19 | 0.032 |
| Cell adhesion molecule 3 | CADM3_HUMAN | 1.18 | 0.037 |
| Secretogranin-1 | SCG1_HUMAN | 1.18 | 0.046 |
| Phosphoinositide-3-kinase-interacting protein 1 | P3IP1_HUMAN | 1.16 | 0.037 |
| Prosaposin receptor GPR37 | GPR37_HUMAN | 1.16 | 0.037 |
| Collagen alpha-1(XVIII) chain | COIA1_HUMAN | 1.12 | 0.048 |
| **Down-regulated proteins** | | | |
| Plasma protease C1 inhibitor | IC1_HUMAN | 0.83 | 0.042 |
| Complement C1s subcomponent | C1S_HUMAN | 0.83 | 0.012 |
| Complement C1r subcomponent | C1R_HUMAN | 0.82 | 0.014 |
| Laminin subunit beta-1 | LAMB1_HUMAN | 0.80 | 0.019 |
| Monocyte differentiation antigen CD14 | CD14_HUMAN | 0.80 | 0.047 |
| Fructose-bisphosphate aldolase A | ALDOA_HUMAN | 0.76 | 0.007 |
| Complement C4-A | CO4A_HUMAN | 0.74 | 0.027 |
| Alpha-1-antichymotrypsin | AACT_HUMAN | 0.74 | 0.016 |
| Complement C1q subcomponent subunit C | C1QC_HUMAN | 0.71 | 0.001 |
| Complement C2 | CO2_HUMAN | 0.71 | 0.009 |
| Transforming growth factor-beta-induced protein ig-h3 | BGH3_HUMAN | 0.69 | 0.002 |
| Scavenger receptor cysteine-rich type 1 protein M130 | C163A_HUMAN | 0.68 | 0.030 |
| Complement C1q subcomponent subunit A | C1QA_HUMAN | 0.67 | 0.013 |
| Complement C1q subcomponent subunit B | C1QB_HUMAN | 0.64 | 0.004 |
| Peptidyl-prolyl cis-trans isomerase A | PPIA_HUMAN | 0.62 | 0.036 |
| Leucine-rich alpha-2-glycoprotein | A2GL_HUMAN | 0.62 | 0.015 |
| Thyroxine-binding globulin | THBG_HUMAN | 0.61 | 0.005 |
| Galectin-3-binding protein | LG3BP_HUMAN | 0.58 | 0.0001 |
| 14-3-3 protein zeta/delta | 1433Z_HUMAN | 0.57 | 0.011 |
| IgGFc-binding protein | FCGBP_HUMAN | 0.42 | 0.018 |

**Supplementary Table 2.** List of significantly regulated proteins in the ALS group vs non-ALS group (no missing values).

| **Protein name** | **Uniprot_name** | **Ratio** | **p-value** |
| --- | --- | --- | --- |
| **Up-regulated proteins** |  |  |  |
| Ganglioside GM2 activator | SAP3_HUMAN | 1,66 | 1,78E-06 |
| Apolipoprotein A-IV | APOA4_HUMAN | 1,61 | 0,006 |
| Clusterin | CLUS_HUMAN | 1,56 | 6,13E-08 |
| Prothrombin | THRB_HUMAN | 1,49 | 0,013 |
| Latent-transforming growth factor beta-binding protein 4 | LTBP4_HUMAN | 1,46 | 0,0003 |
| Pyruvate kinase PKM | KPYM_HUMAN | 1,37 | 0,001 |
| Amyloid-like protein 2 | APLP2_HUMAN | 1,36 | 0,001 |
| Neurosecretory protein VGF | VGF_HUMAN | 1,35 | 0,012 |
| Adhesion G protein-coupled receptor L1 | AGRL1_HUMAN | 1,35 | 0,002 |
| Galectin-1 | LEG1_HUMAN | 1,33 | 0,025 |
| Glucosidase 2 subunit beta | GLU2B_HUMAN | 1,32 | 0,001 |
| Amyloid-beta precursor protein | A4_HUMAN | 1,32 | 0,002 |
| Brevican core protein | PGCB_HUMAN | 1,32 | 0,001 |
| Neurocan core protein | NCAN_HUMAN | 1,32 | 0,006 |
| Vitamin K-dependent protein S | PROS_HUMAN | 1,31 | 0,001 |
| Semaphorin-7A | SEM7A_HUMAN | 1,31 | 0,034 |
| Immunoglobulin superfamily member 8 | IGSF8_HUMAN | 1,31 | 0,003 |
| Endonuclease domain-containing 1 protein | ENDD1_HUMAN | 1,31 | 0,002 |
| CD59 glycoprotein | CD59_HUMAN | 1,29 | 0,003 |
| Neuroendocrine protein 7B2 | 7B2_HUMAN | 1,29 | 0,002 |
| Kallikrein-6 | KLK6_HUMAN | 1,29 | 0,007 |
| Lymphocyte antigen 6H | LY6H_HUMAN | 1,29 | 0,020 |
| Calreticulin | CALR_HUMAN | 1,29 | 0,004 |
| Protein kinase C-binding protein NELL2 | NELL2_HUMAN | 1,28 | 0,012 |
| Cadherin-13 | CAD13_HUMAN | 1,28 | 0,008 |
| ProSAAS | PCS1N_HUMAN | 1,27 | 0,001 |
| Opioid-binding protein/cell adhesion molecule | OPCM_HUMAN | 1,27 | 0,016 |
| Amyloid-like protein 1 | APLP1_HUMAN | 1,27 | 0,002 |
| Apolipoprotein E | APOE_HUMAN | 1,27 | 0,014 |
| Extracellular matrix protein 1 | ECM1_HUMAN | 1,24 | 6,74E-05 |
| Mimecan | MIME_HUMAN | 1,23 | 0,010 |
| Neurofascin | NFASC_HUMAN | 1,23 | 0,016 |
| Secretogranin-2 | SCG2_HUMAN | 1,23 | 0,015 |
| EGF-containing fibulin-like extracellular matrix protein 1 | FBLN3_HUMAN | 1,23 | 0,011 |
| Dystroglycan | DAG1_HUMAN | 1,22 | 0,000 |
| CCN family member 3 | CCN3_HUMAN | 1,22 | 0,031 |
| NPC intracellular cholesterol transporter 2 | NPC2_HUMAN | 1,21 | 0,011 |
| Protein FAM3C | FAM3C_HUMAN | 1,21 | 0,017 |
| Cadherin-2 | CADH2_HUMAN | 1,21 | 0,007 |
| Endoplasmic reticulum chaperone BiP | BIP_HUMAN | 1,21 | 0,002 |
| Insulin-like growth factor-binding protein 2 | IBP2_HUMAN | 1,20 | 0,025 |
| Collagen alpha-1(III) chain | CO3A1_HUMAN | 1,19 | 0,038 |
| Dickkopf-related protein 3 | DKK3_HUMAN | 1,19 | 0,032 |
| Cell adhesion molecule 3 | CADM3_HUMAN | 1,18 | 0,037 |
| Secretogranin-1 | SCG1_HUMAN | 1,18 | 0,046 |
| Phosphoinositide-3-kinase-interacting protein 1 | P3IP1_HUMAN | 1,16 | 0,037 |
| **Down-regulated proteins** |  |  |  |
| Plasma protease C1 inhibitor | IC1_HUMAN | 0,83 | 0,042 |
| Complement C1s subcomponent | C1S_HUMAN | 0,83 | 0,012 |
| Complement C1r subcomponent | C1R_HUMAN | 0,82 | 0,014 |
| Monocyte differentiation antigen CD14 | CD14_HUMAN | 0,80 | 0,047 |
| Fructose-bisphosphate aldolase A | ALDOA_HUMAN | 0,76 | 0,007 |
| Alpha-1-antichymotrypsin | AACT_HUMAN | 0,74 | 0,016 |
| Complement C1q subcomponent subunit C | C1QC_HUMAN | 0,71 | 0,001 |
| Complement C2 | CO2_HUMAN | 0,71 | 0,009 |
| Scavenger receptor cysteine-rich type 1 protein M130 | C163A_HUMAN | 0,68 | 0,030 |
| Leucine-rich alpha-2-glycoprotein | A2GL_HUMAN | 0,62 | 0,015 |
| Galectin-3-binding protein | LG3BP_HUMAN | 0,58 | 0,0001 |
| IgGFc-binding protein | FCGBP_HUMAN | 0,42 | 0,018 |
|  |  |  |  |

**Supplementary Tables 3 A-D.** A. Coefficients of variation of the analysed proteins in ALS (20% missing values), B. Coefficients of variation of the analysed proteins in non-ALS group (20% missing values), C. Coefficients of variation of the analysed proteins in ALS (no missing values), D. Coefficients of variation of the analysed proteins in non-ALS group (no missing values).

| **A Coefficients of variation of the analysed proteins in ALS (20% missing values)** | | | | |
| --- | --- | --- | --- | --- |
| **Protein** | **Mean** | **Std** | **N** | **CV** |
| **CLUS** | 15667950000 | 3140494848 | 26 | 0,200440699 |
| **SAP3** | 840925384,6 | 241229704,8 | 26 | 0,286862199 |
| **LG3BP** | 201387692,3 | 65959717,91 | 26 | 0,327526063 |
| **ECM1** | 827839230,8 | 141913397,5 | 26 | 0,171426277 |
| **DAG1** | 596559230,8 | 105591037,1 | 26 | 0,177000089 |
| **RGMA** | 38453782,61 | 8022960,364 | 23 | 0,208639042 |
| **FGFR2** | 41991360 | 7925611,322 | 25 | 0,188743859 |
| **LTBP4** | 114177769,2 | 36350546,16 | 26 | 0,318367984 |
| **LYNX1** | 193850565,2 | 63020410,68 | 23 | 0,325097895 |
| **MA1A1** | 33976080 | 6238947,827 | 25 | 0,183627653 |
| **PROS** | 334903076,9 | 77620713,77 | 26 | 0,23177068 |
| **DAF** | 86857320 | 23491814,85 | 25 | 0,270464422 |
| **GLU2B** | 61691653,85 | 17266574,62 | 26 | 0,279885099 |
| **VTM2B** | 40789960 | 11588324,56 | 25 | 0,284097473 |
| **APLP2** | 307261923,1 | 86110272,47 | 26 | 0,280250386 |
| **SEPP1** | 99030320 | 26410203,71 | 25 | 0,266688058 |
| **KPYM** | 124329692,3 | 33028046,7 | 26 | 0,265648906 |
| **DNER** | 47577083,33 | 16609601,42 | 24 | 0,349109282 |
| **PCS1N** | 914526923,1 | 216147189,2 | 26 | 0,236348634 |
| **PGCB** | 1135550769 | 307745446,2 | 26 | 0,271009852 |
| **C1QC** | 157097615,4 | 31025611,37 | 26 | 0,197492567 |
| **BIP** | 49122923,08 | 8700736,01 | 26 | 0,177121707 |
| **7B2** | 655847307,7 | 168936714,8 | 26 | 0,257585436 |
| **ENDD1** | 238442230,8 | 57702854,72 | 26 | 0,241999307 |
| **APLP1** | 3410076923 | 843447730,8 | 26 | 0,247339796 |
| **BGH3** | 69587769,23 | 33786364,41 | 26 | 0,485521591 |
| **AGRL1** | 114354538,5 | 33582727,87 | 26 | 0,293672016 |
| **A4** | 2738534615 | 807625869,3 | 26 | 0,294911689 |
| **OLFL3** | 28065400 | 6434623,131 | 25 | 0,229272454 |
| **IGSF8** | 159288269,2 | 40832223,57 | 26 | 0,256341686 |
| **HYOU1** | 43378040 | 11122762,08 | 25 | 0,256414584 |
| **CD59** | 875697307,7 | 233372370,5 | 26 | 0,266498901 |
| **C1QB** | 118783846,2 | 34369129,76 | 26 | 0,289341782 |
| **CALR** | 96243269,23 | 26040718,74 | 26 | 0,270571843 |
| **GFRA2** | 56371200 | 20647588,56 | 25 | 0,366279032 |
| **PCDH1** | 42661346,15 | 11332240,85 | 26 | 0,265632519 |
| **CAD15** | 20066733,33 | 8736323,227 | 24 | 0,435363498 |
| **THBG** | 45359708,33 | 28672546,45 | 24 | 0,632114877 |
| **APOA4** | 2841111538 | 1415666028 | 26 | 0,498278934 |
| **NCAN** | 559171538,5 | 175950928,2 | 26 | 0,314663598 |
| **NRX2A** | 111001000 | 32075931,46 | 26 | 0,288969752 |
| **CADH2** | 431534230,8 | 93311621,15 | 26 | 0,216232258 |
| **GPC1** | 26804347,83 | 6643381,078 | 23 | 0,247847145 |
| **ALDOA** | 75330884,62 | 25653855,42 | 26 | 0,340548973 |
| **KLK6** | 3216843077 | 999025420 | 26 | 0,310560819 |
| **NECT1** | 65903043,48 | 15785059,93 | 23 | 0,239519438 |
| **CAD13** | 480084615,4 | 155113073 | 26 | 0,32309528 |
| **CO2** | 195132500 | 72482132,35 | 26 | 0,371450847 |
| **MIME** | 1925741154 | 502123453,8 | 26 | 0,260742963 |
| **TICN3** | 33236333,33 | 7002007,53 | 24 | 0,210673285 |
| **FBLN3** | 939973846,2 | 209386458,4 | 26 | 0,222757749 |
| **NPC2** | 2009818462 | 555519923 | 26 | 0,276403035 |
| **1433Z** | 57018166,67 | 28191836,88 | 24 | 0,494436046 |
| **VGF** | 2880532308 | 993016973,8 | 26 | 0,344733844 |
| **HABP2** | 55767384,62 | 28194564,02 | 26 | 0,505574436 |
| **TNR21** | 62249083,33 | 15397503,34 | 24 | 0,247353094 |
| **C1S** | 637388076,9 | 116082703,7 | 26 | 0,18212249 |
| **NELL2** | 959877307,7 | 303715094,8 | 26 | 0,316410329 |
| **C1QA** | 257765160 | 79672008,9 | 25 | 0,309087578 |
| **THRB** | 1739792692 | 855518116,9 | 26 | 0,491735665 |
| **APOE** | 21583307692 | 6403679603 | 26 | 0,296695933 |
| **C1R** | 729235000 | 118371041 | 26 | 0,162322216 |
| **OMGP** | 119120615,4 | 39662392,35 | 26 | 0,332959935 |
| **FGFR1** | 71402360 | 20045262,08 | 25 | 0,280736688 |
| **A2GL** | 214810538,5 | 120459817,9 | 26 | 0,560772385 |
| **SCG2** | 980983461,5 | 259826812,9 | 26 | 0,264863602 |
| **OPCM** | 370428846,2 | 111845918,2 | 26 | 0,301936308 |
| **NFASC** | 245228846,2 | 68617120,39 | 26 | 0,27980852 |
| **AACT** | 3158588462 | 1267623001 | 26 | 0,401325787 |
| **FAM3C** | 999401923,1 | 246983243,8 | 26 | 0,247131047 |
| **CGRE1** | 113615320 | 28130470,93 | 25 | 0,247593995 |
| **FCGBP** | 363386923,1 | 182890309,6 | 26 | 0,503293591 |
| **LAMB1** | 26259434,78 | 5550835,628 | 23 | 0,211384429 |
| **LY6H** | 180395423,1 | 63783750,67 | 26 | 0,353577433 |
| **NTRK2** | 52726782,61 | 14205384,32 | 23 | 0,269414965 |
| **LEG1** | 64098500 | 28352989,12 | 26 | 0,442334674 |
| **IBP2** | 987683846,2 | 261362236,3 | 26 | 0,264621354 |
| **RGMB** | 62487807,69 | 16732550,89 | 26 | 0,267773051 |
| **NRN1** | 33932409,09 | 10535288,4 | 22 | 0,310478645 |
| **CO4A** | 298799960 | 142259658,5 | 25 | 0,476103339 |
| **C163A** | 130569153,8 | 43009984,96 | 26 | 0,329403873 |
| **CCN3** | 107826846,2 | 31126444,12 | 26 | 0,288670635 |
| **DKK3** | 11126138462 | 3451143455 | 26 | 0,3101834 |
| **RARR2** | 119159080 | 33940598,96 | 25 | 0,284834349 |
| **SEM7A** | 345234230,8 | 141191684,8 | 26 | 0,408973596 |
| **PPIA** | 49911739,13 | 48248224,77 | 23 | 0,96667088 |
| **P3IP1** | 299339230,8 | 76449652,71 | 26 | 0,255394699 |
| **GPR37** | 50819458,33 | 12381790,4 | 24 | 0,243642707 |
| **CADM3** | 356475384,6 | 81418234,28 | 26 | 0,228397914 |
| **CO3A1** | 55619307,69 | 14505254,94 | 26 | 0,260795316 |
| **IC1** | 775885769,2 | 243135260,3 | 26 | 0,313364763 |
| **SCG1** | 6162880769 | 1552463040 | 26 | 0,251905415 |
| **CD14** | 754726923,1 | 217956669 | 26 | 0,288788782 |
| **COIA1** | 153743846,2 | 25107567,64 | 26 | 0,163307789 |
| **FRS1L** | 13983330,43 | 3692500,034 | 23 | 0,26406442 |
| **ECM2** | 22931458,33 | 5886060,364 | 24 | 0,256680595 |
| **SPRL1** | 3092846154 | 713406539,5 | 26 | 0,230663442 |
| **VSIG4** | 72990782,61 | 42222081,46 | 23 | 0,578457717 |
| **AGRL3** | 26025296 | 9190952,084 | 25 | 0,353154565 |
| **IBP3** | 30323807,69 | 11584901,49 | 26 | 0,382039802 |
| **FIBB** | 279845291,3 | 543064820,8 | 23 | 1,940589453 |
| **SHSA5** | 60538360 | 12953987,47 | 25 | 0,213979822 |
| **NPTX1** | 337731153,8 | 112678530,1 | 26 | 0,333633806 |
| **SAP** | 1080689346 | 391409241,7 | 26 | 0,362184788 |
| **PGBM** | 441346923,1 | 101706432,4 | 26 | 0,230445545 |
| **NEGR1** | 476078461,5 | 136745120,3 | 26 | 0,287232319 |
| **IGF2** | 496684615,4 | 188841311,9 | 26 | 0,380203667 |
| **CADM4** | 347168076,9 | 86235689,74 | 26 | 0,248397521 |
| **CADH6** | 18810800 | 4224895,886 | 25 | 0,224599479 |
| **ACTG** | 167281807,7 | 100533735,4 | 26 | 0,600984272 |
| **CO8G** | 64278576,92 | 32831082,3 | 26 | 0,510762432 |
| **TICN1** | 92833576,92 | 26979497,29 | 26 | 0,290622188 |
| **ENOA** | 17302904,76 | 9021039,529 | 21 | 0,521359833 |
| **APOA2** | 1829016923 | 1251159878 | 26 | 0,684061401 |
| **CADM2** | 88636307,69 | 27234231,03 | 26 | 0,307258185 |
| **EFNB2** | 46724125 | 14628240,18 | 24 | 0,313076814 |
| **CO3** | 677974230,8 | 266719360,4 | 26 | 0,393406339 |
| **FHR2** | 39044960 | 21100321,26 | 25 | 0,540410882 |
| **ICOSL** | 51067291,67 | 13064601,09 | 24 | 0,255831094 |
| **CADH4** | 54943375 | 18294212,92 | 24 | 0,332964856 |
| **NPTXR** | 771131923,1 | 231431304,8 | 26 | 0,300118952 |
| **ALDOC** | 68155769,23 | 16649196,07 | 26 | 0,244281537 |
| **CART** | 35129000 | 11101440,03 | 22 | 0,316019244 |
| **LDHB** | 69978400 | 23408643,12 | 25 | 0,334512408 |
| **A1BG** | 3823507692 | 1577806110 | 26 | 0,412659327 |
| **EPHA4** | 154993923,1 | 61487970,42 | 26 | 0,396712137 |
| **BTD** | 127373076,9 | 42443687,63 | 26 | 0,333223383 |
| **VTNC** | 754496923,1 | 406232799,3 | 26 | 0,538415449 |
| **MMP2** | 158395730,8 | 42678498,34 | 26 | 0,269442226 |
| **FMOD** | 29448000 | 11368686,22 | 22 | 0,386059706 |
| **LTBP2** | 45819000 | 15312842,29 | 26 | 0,334202892 |
| **PLXB2** | 49717307,69 | 13330227,48 | 26 | 0,268120462 |
| **CBPE** | 500928846,2 | 129424300 | 26 | 0,258368631 |
| **CD166** | 219706846,2 | 62777346,7 | 26 | 0,285732319 |
| **KAIN** | 55176492 | 38016791,31 | 25 | 0,689003413 |
| **VASN** | 65509653,85 | 13568019,55 | 26 | 0,207114811 |
| **QSOX1** | 40876692,31 | 8174259,735 | 26 | 0,19997361 |
| **ACBP** | 51359454,55 | 15087821,76 | 22 | 0,29376912 |
| **C1RL** | 23964000 | 8013613,406 | 24 | 0,334402162 |
| **FBLN1** | 1291078462 | 249422677,7 | 26 | 0,193189403 |
| **GOLM1** | 83730346,15 | 23354892,14 | 26 | 0,278929841 |
| **PLTP** | 223211038,5 | 127967988,5 | 26 | 0,573304929 |
| **ANGT** | 5044723077 | 2878641886 | 26 | 0,570624362 |
| **OAF** | 37902200 | 9917955,892 | 25 | 0,261672301 |
| **CP089** | 63064324 | 31558973,5 | 25 | 0,500425145 |
| **BASP1** | 39506640 | 12799780,37 | 25 | 0,323990609 |
| **CYTC** | 19299557692 | 7080051376 | 26 | 0,366850448 |
| **L1CAM** | 68063961,54 | 29030794,95 | 26 | 0,426522264 |
| **R4RL2** | 37023269,23 | 13937159,63 | 26 | 0,376443246 |
| **T132A** | 74613208,33 | 19520975,82 | 24 | 0,261628956 |
| **HBB** | 22169526462 | 66162863236 | 26 | 2,984405795 |
| **NCHL1** | 1664258846 | 523794963,1 | 26 | 0,314731668 |
| **PCOC1** | 622424230,8 | 166297827,7 | 26 | 0,267177625 |
| **SBP1** | 42335040 | 36625862,74 | 25 | 0,865142982 |
| **VTDB** | 10294692308 | 4710838696 | 26 | 0,457598785 |
| **CERU** | 6254203846 | 1802220396 | 26 | 0,288161441 |
| **ALBU** | 440193269,2 | 251985728,6 | 26 | 0,572443393 |
| **HBA** | 8694994374 | 24186941555 | 23 | 2,781708707 |
| **RNAS4** | 48584000 | 12718930,77 | 26 | 0,261792581 |
| **CO4B** | 13829553846 | 4697730405 | 26 | 0,339687777 |
| **ITIH1** | 307721769,2 | 208833093,9 | 26 | 0,678642575 |
| **APOH** | 6793292308 | 2913666811 | 26 | 0,428903495 |
| **SCRG1** | 191824083,3 | 78230312,99 | 24 | 0,407823208 |
| **KLKB1** | 17065881,82 | 7918606,499 | 22 | 0,464002188 |
| **SEZ6** | 104150615,4 | 34557600,7 | 26 | 0,331804095 |
| **NRCAM** | 2978023077 | 908610919,3 | 26 | 0,305105399 |
| **PRRT3** | 27889340 | 6599941,791 | 25 | 0,236647471 |
| **NBL1** | 453844076,9 | 243388257,5 | 26 | 0,536281666 |
| **NID1** | 61176920 | 23698412,19 | 25 | 0,387375046 |
| **LAMA2** | 27606904,76 | 8604146,43 | 21 | 0,311666465 |
| **NCAM2** | 280107769,2 | 85989179,65 | 26 | 0,306986057 |
| **CA2D1** | 460748076,9 | 161679556,3 | 26 | 0,350906633 |
| **COMP** | 25214648 | 13876801,45 | 25 | 0,550346824 |
| **ISLR** | 52988653,85 | 6705116,172 | 26 | 0,126538715 |
| **ITIH4** | 812021923,1 | 401722674,2 | 26 | 0,494719001 |
| **TIMP1** | 493032307,7 | 115476580,2 | 26 | 0,234217065 |
| **FIBA** | 720139615,4 | 653372337,2 | 26 | 0,907285647 |
| **FETUB** | 40188956,52 | 22738568,62 | 23 | 0,565791466 |
| **TICN2** | 92889695,65 | 32591448,95 | 23 | 0,350861834 |
| **PTPRG** | 64457920 | 27404748,71 | 25 | 0,425157199 |
| **PLMN** | 2035107692 | 848075402,7 | 26 | 0,416722617 |
| **LYSC** | 135158120 | 92872328,36 | 25 | 0,687138356 |
| **AMD** | 274490769,2 | 77195532,3 | 26 | 0,281231797 |
| **SUSD5** | 71566840 | 26089668,24 | 25 | 0,364549675 |
| **MEGF8** | 222268230,8 | 89457200,42 | 26 | 0,402474074 |
| **MASP1** | 28045230,77 | 8759945,618 | 26 | 0,312350634 |
| **RTN4R** | 76505181,82 | 29142507,42 | 22 | 0,380922007 |
| **CSPG2** | 166226153,8 | 37291238,55 | 26 | 0,22434038 |
| **KNG1** | 838990769,2 | 446133754,9 | 26 | 0,531750493 |
| **K22E** | 1074037423 | 940046355,2 | 26 | 0,875245438 |
| **AFAM** | 1492255769 | 712611528,6 | 26 | 0,477539805 |
| **IPSP** | 24577458,33 | 11798640,36 | 24 | 0,480059419 |
| **CAB45** | 33593523,81 | 7349581,026 | 21 | 0,218779699 |
| **CH3L1** | 1069154615 | 711721517,3 | 26 | 0,665686241 |
| **PTGDS** | 45277192308 | 10353755763 | 26 | 0,228674863 |
| **TIMP2** | 298936153,8 | 78586968,24 | 26 | 0,262888805 |
| **VCAM1** | 43154320 | 19520659,73 | 25 | 0,452345437 |
| **ICAM5** | 47620208,33 | 33621389,4 | 24 | 0,706031968 |
| **PEDF** | 5289207692 | 1175107951 | 26 | 0,222170884 |
| **K2C6A** | 149471750 | 146359264,9 | 24 | 0,979176767 |
| **COL12** | 39652869,57 | 7632836,071 | 23 | 0,192491392 |
| **NEUS** | 48954009,52 | 18678394,38 | 21 | 0,381549838 |
| **CNTN2** | 381446923,1 | 88270452,49 | 26 | 0,231409528 |
| **PMGT1** | 25787666,67 | 9842102,332 | 24 | 0,381659282 |
| **SODC** | 1504302692 | 468825316 | 26 | 0,311656237 |
| **CSF1** | 60845040 | 14853341,24 | 25 | 0,244117536 |
| **HEMO** | 10973573077 | 3903557074 | 26 | 0,355723432 |
| **GOLI4** | 20483304,35 | 6219651,492 | 23 | 0,303644929 |
| **HRG** | 1219829615 | 919953233,9 | 26 | 0,754165354 |
| **SLIK1** | 49285000 | 17710238,84 | 23 | 0,359343387 |
| **SCG3** | 2285752692 | 731540189,6 | 26 | 0,320043455 |
| **IBP5** | 23403478,26 | 8865456,42 | 23 | 0,378809351 |
| **CO7** | 879655384,6 | 285602923,4 | 26 | 0,324675922 |
| **ITIH2** | 340827692,3 | 195003804,6 | 26 | 0,572147772 |
| **AMBP** | 894200000 | 396542097,1 | 26 | 0,443460185 |
| **ITIH5** | 32612461,54 | 9817816,365 | 26 | 0,301044935 |
| **NAR3** | 35187791,67 | 12847816,99 | 24 | 0,365121435 |
| **IL6RB** | 56542875 | 27082503,29 | 24 | 0,478972873 |
| **K1C10** | 3121411538 | 2948797153 | 26 | 0,944699895 |
| **CMGA** | 2974184231 | 1309018184 | 26 | 0,440126799 |
| **SE6L2** | 118586038,5 | 34271553,5 | 26 | 0,289001589 |
| **LSAMP** | 1020612308 | 233858345,1 | 26 | 0,229135337 |
| **CO6A1** | 462909615,4 | 104320704 | 26 | 0,225358689 |
| **TTHY** | 1480356154 | 562104111,5 | 26 | 0,379708701 |
| **TPP1** | 42639375 | 17799902,31 | 24 | 0,417452233 |
| **PON1** | 70783681,82 | 48814817,27 | 22 | 0,689633769 |
| **NID2** | 22829269,23 | 7149793,488 | 26 | 0,313185386 |
| **K2C1** | 4401939231 | 3734964400 | 26 | 0,848481591 |
| **CNTP4** | 37282730,77 | 18815048,25 | 26 | 0,504658534 |
| **LUM** | 551799230,8 | 163795128,9 | 26 | 0,29683827 |
| **GPX3** | 45495360 | 10183730,89 | 25 | 0,223841088 |
| **CO9** | 475556153,8 | 208258736,1 | 26 | 0,437926698 |
| **CSTN1** | 2025184615 | 718291707,1 | 26 | 0,354679619 |
| **GELS** | 7137653846 | 1359614370 | 26 | 0,190484773 |
| **CD44** | 335618538,5 | 125813091,4 | 26 | 0,374869314 |
| **CATB** | 71714884,62 | 22273565,04 | 26 | 0,310584967 |
| **TETN** | 1373492308 | 339866222 | 26 | 0,24744676 |
| **CATZ** | 46492217,39 | 15839924,87 | 23 | 0,340700568 |
| **PTPRZ** | 217702692,3 | 40063336,81 | 26 | 0,184027751 |
| **CO5** | 42276296 | 36220185,08 | 25 | 0,856749255 |
| **CO6A3** | 46796388,46 | 21043363,49 | 26 | 0,449679221 |
| **CSF1R** | 217921692,3 | 114716558,9 | 26 | 0,526411839 |
| **C99L2** | 74953520 | 56640746,01 | 25 | 0,755678266 |
| **ADA22** | 88368173,91 | 24658997,85 | 23 | 0,279048403 |
| **VAS1** | 329101153,8 | 85032774,92 | 26 | 0,258378842 |
| **NEO1** | 188356076,9 | 61901245 | 26 | 0,32863949 |
| **FINC** | 2068742308 | 443310471,9 | 26 | 0,214289847 |
| **SE6L1** | 152431730,8 | 73034295,82 | 26 | 0,479127905 |
| **RNAS1** | 762431153,8 | 278935859 | 26 | 0,365850553 |
| **SPIT2** | 25840652,17 | 5285958,489 | 23 | 0,204559794 |
| **NUCB1** | 201216230,8 | 50511464,55 | 26 | 0,251030766 |
| **NPDC1** | 168933708,3 | 71877084,31 | 24 | 0,425475087 |
| **UFO** | 49667076,92 | 11473350,42 | 26 | 0,231005147 |
| **ZA2G** | 2846789231 | 1537054886 | 26 | 0,539925777 |
| **PTPR2** | 79848000 | 30587665,47 | 26 | 0,383073658 |
| **OSTP** | 3656888462 | 1255286343 | 26 | 0,343266237 |
| **LRC4B** | 38691333,33 | 16032253,73 | 21 | 0,414362917 |
| **CO1A2** | 114304038,5 | 28785403,44 | 26 | 0,251831902 |
| **PTPRN** | 20160476,19 | 5813233,503 | 21 | 0,288348026 |
| **FA12** | 248029160 | 123340054,3 | 25 | 0,497280458 |
| **MUC18** | 281231153,8 | 58921911,12 | 26 | 0,209514168 |
| **SFRP4** | 14447869,57 | 3908466,018 | 23 | 0,270521962 |
| **FSTL4** | 30049808,33 | 17849845,6 | 24 | 0,594008634 |
| **CATL1** | 65696200 | 17883311,21 | 25 | 0,272212262 |
| **ATRN** | 37863000 | 10658645,76 | 26 | 0,281505579 |
| **FA5** | 120618615,4 | 35352757,79 | 26 | 0,293095371 |
| **FBN1** | 8995654,167 | 2694313,608 | 24 | 0,299512805 |
| **K1C16** | 57273075 | 55857592,95 | 24 | 0,975285384 |
| **IBP4** | 157186923,1 | 41660996,16 | 26 | 0,265041107 |
| **MOG** | 49422440 | 13928284,29 | 25 | 0,281821057 |
| **AATC** | 56438307,69 | 30226525,58 | 26 | 0,535567539 |
| **LAMC1** | 15411687,5 | 5719028,296 | 24 | 0,371083848 |
| **AGRIN** | 66066846,15 | 24903160,73 | 26 | 0,376938846 |
| **K1C9** | 3164421538 | 2715895556 | 26 | 0,858259724 |
| **PEBP4** | 183062423,1 | 60694817,64 | 26 | 0,331552574 |
| **PEBP1** | 312963846,2 | 85448748,53 | 26 | 0,273030734 |
| **FHR1** | 104300600 | 46567228,06 | 25 | 0,446471334 |
| **CBPQ** | 34072791,67 | 7181501,914 | 24 | 0,210769402 |
| **PRDX1** | 37075833,33 | 48549850,08 | 24 | 1,309474278 |
| **ANT3** | 4941676923 | 2131845215 | 26 | 0,431401172 |
| **NCAM1** | 1407865385 | 254978104,1 | 26 | 0,181109719 |
| **CRAC1** | 321940115,4 | 102134279,7 | 26 | 0,317246204 |
| **LRP1** | 34203880 | 13455150,41 | 25 | 0,393380821 |
| **NTRI** | 303261923,1 | 69502953,55 | 26 | 0,22918457 |
| **CNDP1** | 1222376538 | 419073739,7 | 26 | 0,342835228 |
| **CO6** | 197868269,2 | 85844834,45 | 26 | 0,433848412 |
| **CO8A** | 102408538,5 | 43164443,1 | 26 | 0,421492619 |
| **PENK** | 423366538,5 | 118384218,2 | 26 | 0,279625826 |
| **B2MG** | 2902569231 | 997608260,9 | 26 | 0,343698352 |
| **PTPRS** | 68323884,62 | 24329798,88 | 26 | 0,356095076 |
| **TPIS** | 383571153,8 | 145329017,9 | 26 | 0,378884117 |
| **CADM1** | 210805384,6 | 59109487,38 | 26 | 0,280398375 |
| **SPON1** | 37914875 | 13047290,38 | 24 | 0,344120622 |
| **K2C5** | 181382658,3 | 171646930,3 | 24 | 0,946324924 |
| **NRX3A** | 160106576,9 | 52959638,79 | 26 | 0,33077741 |
| **CSTN3** | 33940541,67 | 7706522,601 | 24 | 0,227059505 |
| **A2AP** | 367469615,4 | 158135662,4 | 26 | 0,430336702 |
| **SHPS1** | 416322307,7 | 139213010 | 26 | 0,334387583 |
| **IBP7** | 901059230,8 | 349568279,9 | 26 | 0,38795261 |
| **THY1** | 908738076,9 | 341736086,5 | 26 | 0,376055648 |
| **CFAD** | 240024384,6 | 93300650,82 | 26 | 0,388713217 |
| **FABP5** | 33314053,85 | 21799250,9 | 26 | 0,654355996 |
| **LAMP2** | 310537360 | 149940938,7 | 25 | 0,482843477 |
| **IBP6** | 1313615385 | 385681734,5 | 26 | 0,293603241 |
| **G3P** | 61181440 | 46091962,18 | 25 | 0,753365108 |
| **K1C14** | 239330680 | 228198829,8 | 25 | 0,953487576 |
| **TYB4** | 130691250 | 50144738,32 | 24 | 0,383688566 |
| **ENPP2** | 2708276923 | 706732509,1 | 26 | 0,260952823 |
| **SIAE** | 21935360 | 7773367,481 | 25 | 0,354376107 |
| **RELN** | 78479357,69 | 62430640,14 | 26 | 0,795503964 |
| **FSTL1** | 104968884,6 | 30614570,74 | 26 | 0,291653768 |
| **ASPG** | 22365958,33 | 9559269,417 | 24 | 0,42740263 |
| **CFAH** | 2522430769 | 551233687,5 | 26 | 0,218532732 |
| **CFAB** | 2715069231 | 1172940048 | 26 | 0,432011101 |
| **LMAN2** | 86436423,08 | 26538154,87 | 26 | 0,30702514 |
| **DCD** | 102527253,8 | 153843971 | 26 | 1,500517816 |
| **FAT2** | 57855480,77 | 28564130,83 | 26 | 0,49371521 |
| **PRDX2** | 579862491,7 | 1886835818 | 24 | 3,253936657 |
| **TRFE** | 64547576 | 97138334,83 | 25 | 1,504910654 |
| **PI16** | 141639520 | 43876601,41 | 25 | 0,309776547 |
| **LYVE1** | 66620520 | 19358982,25 | 25 | 0,290585877 |
| **APOD** | 1808034231 | 803034472 | 26 | 0,444147825 |
| **CO8B** | 51951923,08 | 30032553,63 | 26 | 0,578083579 |
| **PRIO** | 648378461,5 | 175032213 | 26 | 0,269953775 |
| **CO1A1** | 173627038,5 | 50481673,47 | 26 | 0,290747766 |
| **PGRP2** | 662941538,5 | 307044928,6 | 26 | 0,463155362 |
| **MDHC** | 45734807,69 | 11360787,89 | 26 | 0,248405721 |
| **EPCR** | 23841000 | 8203363,545 | 23 | 0,344086387 |
| **NRX1A** | 110823923,1 | 48519789,78 | 26 | 0,437809711 |
| **PARK7** | 30645538,46 | 20358586,58 | 26 | 0,664324649 |
| **LCAT** | 44344076,92 | 13809985,52 | 26 | 0,311427962 |
| **SODE** | 707981923,1 | 205777719,2 | 26 | 0,290653917 |
| **PPIB** | 42341269,23 | 10022695,5 | 26 | 0,236712212 |
| **FETUA** | 3035800000 | 1662429456 | 26 | 0,547608359 |
| **CNTN1** | 1624816538 | 347758117,2 | 26 | 0,214029159 |
| **SPRC** | 450965384,6 | 102595697,7 | 26 | 0,227502379 |
| **WFKN2** | 107094653,8 | 40956151,24 | 26 | 0,382429466 |
| **OMD** | 192186384,6 | 66267284,48 | 26 | 0,344807384 |
| **HEP2** | 182906807,7 | 72759462,48 | 26 | 0,397795267 |
| **CFAI** | 595407692,3 | 245731630,7 | 26 | 0,412711548 |
| **RET4** | 988023846,2 | 354745781,7 | 26 | 0,359045769 |
| **B4GA1** | 2619773077 | 741878449,1 | 26 | 0,283184241 |
| **RNT2** | 216714423,1 | 62971219,34 | 26 | 0,290572351 |
| **RNAS6** | 90716000 | 22398161,09 | 25 | 0,246904197 |
| **CATD** | 362287307,7 | 95562551,27 | 26 | 0,263775598 |
| **TGON2** | 41466653,85 | 13653824,75 | 26 | 0,329272403 |
| **PTPRD** | 27357020,83 | 11267408,94 | 24 | 0,411865349 |
| **EFNB1** | 20520934,78 | 4783200,584 | 23 | 0,233088825 |

| **B Coefficients of variation of the analysed proteins in non-ALS group (20% missing values)** | | | | |
| --- | --- | --- | --- | --- |
| **Protein** | **Mean** | **Std** | **N** | **CV** |
| **CLUS** | 10042579167 | 3066685006 | 24 | 0,305368268 |
| **SAP3** | 505429833,3 | 189261132,7 | 24 | 0,3744558 |
| **LG3BP** | 346428333,3 | 125522972,1 | 24 | 0,362334601 |
| **ECM1** | 666598333,3 | 116840967,4 | 24 | 0,175279417 |
| **DAG1** | 489676666,7 | 77642302,55 | 24 | 0,158558306 |
| **RGMA** | 29074300 | 7463500,372 | 20 | 0,256704387 |
| **FGFR2** | 33068952,38 | 7481160,181 | 21 | 0,226229126 |
| **LTBP4** | 78281250 | 28306759,13 | 24 | 0,36160331 |
| **LYNX1** | 121209157,9 | 58477883,5 | 19 | 0,482454334 |
| **MA1A1** | 27850260,87 | 5072218,685 | 23 | 0,182124638 |
| **PROS** | 254836666,7 | 75721101,28 | 24 | 0,297135818 |
| **DAF** | 62457565,22 | 22125794,65 | 23 | 0,354253237 |
| **GLU2B** | 46743625 | 10451875,72 | 24 | 0,223600025 |
| **VTM2B** | 29959695,65 | 8829033,173 | 23 | 0,294697025 |
| **APLP2** | 226417375 | 72387887,35 | 24 | 0,319709949 |
| **SEPP1** | 75643541,67 | 20283905,43 | 24 | 0,268151186 |
| **KPYM** | 91015408,33 | 35196396,03 | 24 | 0,386708104 |
| **DNER** | 33273347,83 | 10973581,67 | 23 | 0,329800948 |
| **PCS1N** | 718375416,7 | 183990015,3 | 24 | 0,256119587 |
| **PGCB** | 862550416,7 | 259256427,8 | 24 | 0,300569593 |
| **C1QC** | 221905666,7 | 92537544,37 | 24 | 0,417012985 |
| **BIP** | 40765208,33 | 8909572,053 | 24 | 0,218558237 |
| **7B2** | 508185416,7 | 141416299,3 | 24 | 0,278276973 |
| **ENDD1** | 182627500 | 61614163,41 | 24 | 0,337376153 |
| **APLP1** | 2690908333 | 714280674,3 | 24 | 0,265442217 |
| **BGH3** | 100807409,1 | 33001455,52 | 22 | 0,327371329 |
| **AGRL1** | 84903050 | 31062812,9 | 24 | 0,365862156 |
| **A4** | 2078841667 | 632023036,8 | 24 | 0,304026539 |
| **OLFL3** | 21776142,86 | 6894720,446 | 21 | 0,316618076 |
| **IGSF8** | 121809958,3 | 43384984,48 | 24 | 0,356169439 |
| **HYOU1** | 34468200 | 6794225,099 | 20 | 0,19711575 |
| **CD59** | 676478666,7 | 219230574,4 | 24 | 0,324076109 |
| **C1QB** | 184186695,7 | 103155571,2 | 23 | 0,56005984 |
| **CALR** | 74842375 | 23750598,82 | 24 | 0,317341597 |
| **GFRA2** | 39078023,81 | 17820350,38 | 21 | 0,456019743 |
| **PCDH1** | 33907545,45 | 8563094,199 | 22 | 0,252542438 |
| **CAD15** | 13839875 | 3820781,535 | 20 | 0,276070523 |
| **THBG** | 74695208,33 | 40130527,95 | 24 | 0,537257059 |
| **APOA4** | 1762817500 | 1203342240 | 24 | 0,682624401 |
| **NCAN** | 424797041,7 | 150883933,2 | 24 | 0,35519064 |
| **NRX2A** | 84626304,35 | 32360063,94 | 23 | 0,382387772 |
| **CADH2** | 357647916,7 | 90116820,94 | 24 | 0,25197077 |
| **GPC1** | 20281107,69 | 6488448,534 | 13 | 0,319925747 |
| **ALDOA** | 99771583,33 | 35678689,73 | 24 | 0,357603724 |
| **KLK6** | 2496860833 | 800922854 | 24 | 0,320771924 |
| **NECT1** | 53132652,17 | 15312844,19 | 23 | 0,28820026 |
| **CAD13** | 375944583,3 | 106507760,8 | 24 | 0,283307076 |
| **CO2** | 275859583,3 | 129845207,1 | 24 | 0,470693117 |
| **MIME** | 1560606250 | 455019158 | 24 | 0,291565639 |
| **TICN3** | 27645600 | 6718302,741 | 20 | 0,243015263 |
| **FBLN3** | 767101166,7 | 250341758 | 24 | 0,326347774 |
| **NPC2** | 1663491667 | 331497016,7 | 24 | 0,199277834 |
| **1433Z** | 100243727,3 | 74569825,42 | 22 | 0,743885203 |
| **VGF** | 2132979167 | 1020178317 | 24 | 0,47828799 |
| **HABP2** | 37799782,61 | 17867192,97 | 23 | 0,472679781 |
| **TNR21** | 51001350 | 12399326,65 | 20 | 0,243117617 |
| **C1S** | 772244166,7 | 234036932,7 | 24 | 0,303060797 |
| **NELL2** | 748767500 | 268451364,2 | 24 | 0,358524327 |
| **C1QA** | 383301904,8 | 225394940,4 | 21 | 0,588035012 |
| **THRB** | 1171229167 | 686716961,7 | 24 | 0,586321602 |
| **APOE** | 17050241667 | 6115726770 | 24 | 0,358688568 |
| **C1R** | 888446250 | 294812171,5 | 24 | 0,331828933 |
| **OMGP** | 92900565,22 | 31703798,41 | 23 | 0,341265937 |
| **FGFR1** | 57042958,33 | 19713251,69 | 24 | 0,345586068 |
| **A2GL** | 344081833,3 | 230226221,8 | 24 | 0,669103101 |
| **SCG2** | 798176666,7 | 253560529,6 | 24 | 0,317674696 |
| **OPCM** | 291859500 | 110306303,1 | 24 | 0,377943165 |
| **NFASC** | 199127475 | 61265302,72 | 24 | 0,307668757 |
| **AACT** | 4254350000 | 1806976050 | 24 | 0,424736105 |
| **FAM3C** | 827699166,7 | 241795966 | 24 | 0,292130252 |
| **CGRE1** | 94617130,43 | 25150646,09 | 23 | 0,265814932 |
| **FCGBP** | 865529583,3 | 1028523715 | 24 | 1,188317228 |
| **LAMB1** | 32664380,95 | 11152459,63 | 21 | 0,341425715 |
| **LY6H** | 140149833,3 | 53031070,48 | 24 | 0,378388395 |
| **NTRK2** | 43886500 | 10898845,97 | 22 | 0,248341653 |
| **LEG1** | 48208750 | 18677098,44 | 24 | 0,387421338 |
| **IBP2** | 821864166,7 | 245506474,4 | 24 | 0,298719039 |
| **RGMB** | 50868304,35 | 18534499,64 | 23 | 0,364362443 |
| **NRN1** | 27101238,1 | 8658115,302 | 21 | 0,319473054 |
| **CO4A** | 401795652,2 | 168848892,3 | 23 | 0,420235738 |
| **C163A** | 193207666,7 | 135843681,5 | 24 | 0,703096745 |
| **CCN3** | 88664125 | 29697040,27 | 24 | 0,334938627 |
| **DKK3** | 9369591667 | 1902425989 | 24 | 0,203042572 |
| **RARR2** | 97827833,33 | 34042223,27 | 24 | 0,347980959 |
| **SEM7A** | 263453958,3 | 121735467,4 | 24 | 0,462074922 |
| **PPIA** | 79863782,61 | 45871961,82 | 23 | 0,574377525 |
| **P3IP1** | 257307916,7 | 60033509,19 | 24 | 0,233313883 |
| **GPR37** | 43819043,48 | 9710081,226 | 23 | 0,221595007 |
| **CADM3** | 301084500 | 101031023,3 | 24 | 0,335557039 |
| **CO3A1** | 46832666,67 | 14614235,05 | 24 | 0,312052165 |
| **IC1** | 937131250 | 302445302,8 | 24 | 0,322735265 |
| **SCG1** | 5223470833 | 1685170526 | 24 | 0,322615093 |
| **CD14** | 942184166,7 | 409924506,1 | 24 | 0,435078959 |
| **COIA1** | 137043869,6 | 32484142,72 | 23 | 0,237034629 |
| **FRS1L** | 11451261,11 | 4261994,314 | 18 | 0,372185585 |
| **ECM2** | 18128423,81 | 9762332,169 | 21 | 0,538509706 |
| **SPRL1** | 2680141667 | 736040184,3 | 24 | 0,274627343 |
| **VSIG4** | 138150062,5 | 147544263,1 | 16 | 1,067999974 |
| **AGRL3** | 21285043,48 | 7069586,47 | 23 | 0,332138691 |
| **IBP3** | 24062714,29 | 9652583,453 | 21 | 0,401142753 |
| **FIBB** | 1352333409 | 2539785571 | 22 | 1,878076481 |
| **SHSA5** | 52527956,52 | 15482554,43 | 23 | 0,294748843 |
| **NPTX1** | 279771708,3 | 97451712,13 | 24 | 0,348325829 |
| **SAP** | 898398750 | 258762471,3 | 24 | 0,288026304 |
| **PGBM** | 386625000 | 99835981,79 | 24 | 0,258224331 |
| **NEGR1** | 401524666,7 | 139228927,9 | 24 | 0,346750622 |
| **IGF2** | 407714583,3 | 134572025,6 | 24 | 0,330064293 |
| **CADM4** | 301133750 | 85973375,6 | 24 | 0,28549897 |
| **CADH6** | 16633409,09 | 3590219,7 | 22 | 0,215843889 |
| **ACTG** | 235753833,3 | 156962443,8 | 24 | 0,665789572 |
| **CO8G** | 85255416,67 | 46802398,76 | 24 | 0,548966864 |
| **TICN1** | 79060217,39 | 24986093,88 | 23 | 0,31603877 |
| **ENOA** | 22405480 | 8729326,24 | 20 | 0,38960675 |
| **APOA2** | 1284167917 | 793933356,7 | 24 | 0,618247307 |
| **CADM2** | 74223045,45 | 27521494,09 | 22 | 0,37079446 |
| **EFNB2** | 39507352,94 | 8968425,147 | 17 | 0,227006481 |
| **CO3** | 551311666,7 | 229200929,7 | 24 | 0,415737492 |
| **FHR2** | 28861083,33 | 13912948,54 | 18 | 0,482066053 |
| **ICOSL** | 44652368,42 | 9932590,51 | 19 | 0,222442635 |
| **CADH4** | 46874700 | 10541936,34 | 20 | 0,224896081 |
| **NPTXR** | 647063666,7 | 275348381,3 | 24 | 0,425535222 |
| **ALDOC** | 84737217,39 | 45841230,39 | 23 | 0,540981068 |
| **CART** | 29543478,26 | 10675374,86 | 23 | 0,36134455 |
| **LDHB** | 59338304,35 | 19798972,6 | 23 | 0,333662595 |
| **A1BG** | 4673541667 | 1980091895 | 24 | 0,423681233 |
| **EPHA4** | 127706412,5 | 54187692,16 | 24 | 0,424314575 |
| **BTD** | 148481291,7 | 47806596,25 | 24 | 0,321970504 |
| **VTNC** | 593455000 | 265956001,9 | 24 | 0,448148557 |
| **MMP2** | 182782160,9 | 60992462,84 | 23 | 0,333689363 |
| **FMOD** | 23556823,53 | 10765181,5 | 17 | 0,456987823 |
| **LTBP2** | 38261625 | 17526717,4 | 24 | 0,458075615 |
| **PLXB2** | 43901727,27 | 11105853,57 | 22 | 0,252970766 |
| **CBPE** | 435687083,3 | 155001073,7 | 24 | 0,35576238 |
| **CD166** | 194263000 | 49098553,49 | 24 | 0,252742692 |
| **KAIN** | 76192375 | 53798011,02 | 24 | 0,706081298 |
| **VASN** | 59475391,3 | 13118166,36 | 23 | 0,220564608 |
| **QSOX1** | 44682416,67 | 8938144,637 | 24 | 0,20003718 |
| **ACBP** | 58734809,52 | 15668709,72 | 21 | 0,266770418 |
| **C1RL** | 28879650 | 12649324,13 | 20 | 0,438001296 |
| **FBLN1** | 1165843333 | 321828244,2 | 24 | 0,276047591 |
| **GOLM1** | 73276083,33 | 25366766,13 | 24 | 0,346180704 |
| **PLTP** | 277904260,9 | 123544405,3 | 23 | 0,444557435 |
| **ANGT** | 6147108333 | 2212998773 | 24 | 0,36000647 |
| **OAF** | 33826684,21 | 7235786,435 | 19 | 0,213907647 |
| **CP089** | 76339434,78 | 29727710,53 | 23 | 0,389414863 |
| **BASP1** | 34363333,33 | 11398886,71 | 24 | 0,33171656 |
| **CYTC** | 16704941667 | 5111396830 | 24 | 0,305981124 |
| **L1CAM** | 57244260,87 | 21698773,22 | 23 | 0,379055872 |
| **R4RL2** | 31697454,55 | 10809052,88 | 22 | 0,341006968 |
| **T132A** | 66028956,52 | 20966321,12 | 23 | 0,31753222 |
| **HBB** | 54541047917 | 90844217657 | 24 | 1,665611885 |
| **NCHL1** | 1451475833 | 514333022,9 | 24 | 0,354351765 |
| **PCOC1** | 555002083,3 | 163218179,9 | 24 | 0,294085707 |
| **SBP1** | 56016173,91 | 28633278,22 | 23 | 0,511160906 |
| **VTDB** | 8652075000 | 3254565740 | 24 | 0,37616014 |
| **CERU** | 7392454167 | 3637766584 | 24 | 0,492091869 |
| **ALBU** | 625982375 | 616247884,2 | 24 | 0,984449257 |
| **HBA** | 19808559913 | 28982078772 | 23 | 1,463108823 |
| **RNAS4** | 43446913,04 | 12798920,22 | 23 | 0,294587563 |
| **CO4B** | 15912037500 | 5758896706 | 24 | 0,36192076 |
| **ITIH1** | 457168458,3 | 498150813,6 | 24 | 1,089643882 |
| **APOH** | 5811683333 | 1926309759 | 24 | 0,331454701 |
| **SCRG1** | 233017083,3 | 123210233,4 | 24 | 0,528760517 |
| **KLKB1** | 13716652,94 | 6980483,108 | 17 | 0,508905718 |
| **SEZ6** | 91281304,35 | 31275952,78 | 23 | 0,342632623 |
| **NRCAM** | 2634830000 | 897263063,5 | 24 | 0,340539262 |
| **PRRT3** | 24957260,87 | 8700242,626 | 23 | 0,348605669 |
| **NBL1** | 372465652,2 | 178829616,3 | 23 | 0,480123778 |
| **NID1** | 71829039,13 | 32187280,52 | 23 | 0,44810958 |
| **LAMA2** | 24391850 | 7048098,006 | 20 | 0,288952991 |
| **NCAM2** | 248104583,3 | 89080953,31 | 24 | 0,35904598 |
| **CA2D1** | 400437166,7 | 168471739 | 24 | 0,420719536 |
| **COMP** | 83905022,73 | 227929354,3 | 22 | 2,71651621 |
| **ISLR** | 49105958,33 | 13797648,62 | 24 | 0,280977077 |
| **ITIH4** | 1002423333 | 635994982,9 | 24 | 0,634457481 |
| **TIMP1** | 620017500 | 495118374 | 24 | 0,798555483 |
| **FIBA** | 1619917500 | 3545981194 | 24 | 2,188988756 |
| **FETUB** | 31443500 | 8544726,183 | 12 | 0,271748571 |
| **TICN2** | 82057238,1 | 22485487,75 | 21 | 0,274021991 |
| **PTPRG** | 55899434,78 | 18046890,71 | 23 | 0,322845674 |
| **PLMN** | 1757008750 | 701749609,1 | 24 | 0,399400179 |
| **LYSC** | 180765625 | 156828122 | 24 | 0,867577129 |
| **AMD** | 243869291,7 | 96853855,51 | 24 | 0,397154783 |
| **SUSD5** | 64066521,74 | 13223440,1 | 23 | 0,206401717 |
| **MEGF8** | 192947458,3 | 77204785,54 | 24 | 0,400133727 |
| **MASP1** | 24359190,48 | 11856891,96 | 21 | 0,486752299 |
| **RTN4R** | 66132523,81 | 26523344,62 | 21 | 0,401063548 |
| **CSPG2** | 152164521,7 | 45230861,12 | 23 | 0,297249718 |
| **KNG1** | 710494583,3 | 295514568,2 | 24 | 0,415927968 |
| **K22E** | 1406476167 | 1041825451 | 24 | 0,740734522 |
| **AFAM** | 1283815833 | 502431532,8 | 24 | 0,39135795 |
| **IPSP** | 28770409,09 | 12482032,81 | 22 | 0,433849681 |
| **CAB45** | 31042700 | 6558883,868 | 20 | 0,21128587 |
| **CH3L1** | 1440008500 | 1445300074 | 24 | 1,003674682 |
| **PTGDS** | 48702958333 | 10693372976 | 24 | 0,21956311 |
| **TIMP2** | 271632916,7 | 89347167,08 | 24 | 0,328926141 |
| **VCAM1** | 49821833,33 | 21144931,05 | 24 | 0,424410939 |
| **ICAM5** | 38132347,83 | 21486073,61 | 23 | 0,563460548 |
| **PEDF** | 5724433333 | 1540412138 | 24 | 0,269094258 |
| **K2C6A** | 207019776,2 | 196066368,6 | 21 | 0,947090042 |
| **COL12** | 37275095,24 | 6555257,858 | 21 | 0,175861599 |
| **NEUS** | 57335095,24 | 29653722,36 | 21 | 0,517200194 |
| **CNTN2** | 346446000 | 135316529,8 | 24 | 0,390584766 |
| **PMGT1** | 22547183,33 | 9088535,743 | 18 | 0,403089628 |
| **SODC** | 1642645000 | 436114894,2 | 24 | 0,265495524 |
| **CSF1** | 56409625 | 14134684,46 | 24 | 0,250572211 |
| **HEMO** | 12176725000 | 4299186747 | 24 | 0,353065931 |
| **GOLI4** | 18262806,67 | 6871649,681 | 15 | 0,376264711 |
| **HRG** | 1462845833 | 744961655,3 | 24 | 0,509255069 |
| **SLIK1** | 42689100 | 24669026,06 | 20 | 0,577876462 |
| **SCG3** | 2080092500 | 703593949,4 | 24 | 0,338251279 |
| **IBP5** | 21081043,48 | 6588991,918 | 23 | 0,312555302 |
| **CO7** | 986452500 | 454507041,9 | 24 | 0,46074904 |
| **ITIH2** | 427009416,7 | 392594945,8 | 24 | 0,919405827 |
| **AMBP** | 796940000 | 287179480,5 | 24 | 0,3603527 |
| **ITIH5** | 29510130,43 | 12193007,52 | 23 | 0,413180401 |
| **NAR3** | 31849285,71 | 9346675,447 | 21 | 0,293465779 |
| **IL6RB** | 50013347,83 | 17302522,94 | 23 | 0,345958103 |
| **K1C10** | 3905927917 | 2731133716 | 24 | 0,699227885 |
| **CMGA** | 2609429583 | 1345941981 | 24 | 0,515799311 |
| **SE6L2** | 106947291,7 | 49696916,95 | 24 | 0,464686073 |
| **LSAMP** | 958085000 | 222723585,2 | 24 | 0,232467459 |
| **CO6A1** | 429976666,7 | 135922624,1 | 24 | 0,316116279 |
| **TTHY** | 1639228333 | 651045882,8 | 24 | 0,397166075 |
| **TPP1** | 48120826,09 | 22738728,96 | 23 | 0,472534052 |
| **PON1** | 59123300 | 30765343,29 | 20 | 0,520359034 |
| **NID2** | 24681190,48 | 6679542,01 | 21 | 0,270632894 |
| **K2C1** | 5316275417 | 3489504980 | 24 | 0,656381528 |
| **CNTP4** | 32868500 | 14365017,28 | 20 | 0,437045113 |
| **LUM** | 509451666,7 | 180301940,5 | 24 | 0,353913732 |
| **GPX3** | 42397210,53 | 13451735,24 | 19 | 0,31727878 |
| **CO9** | 426836666,7 | 188091213,8 | 24 | 0,440663206 |
| **CSTN1** | 1860897500 | 639462141,9 | 24 | 0,343631039 |
| **GELS** | 6847004167 | 1030957520 | 24 | 0,150570599 |
| **CD44** | 307586833,3 | 116396533 | 24 | 0,378418451 |
| **CATB** | 80868291,67 | 53338971,43 | 24 | 0,659578313 |
| **TETN** | 1289072917 | 417368204,3 | 24 | 0,32377393 |
| **CATZ** | 43199458,33 | 13066884,64 | 24 | 0,302477974 |
| **PTPRZ** | 209023333,3 | 39471077,54 | 24 | 0,188835748 |
| **CO5** | 52518347,83 | 55186781,83 | 23 | 1,050809557 |
| **CO6A3** | 51438550 | 19942735,49 | 20 | 0,387700188 |
| **CSF1R** | 195497708,3 | 93213255,37 | 24 | 0,476799734 |
| **C99L2** | 65098250 | 31009169,22 | 24 | 0,476344129 |
| **ADA22** | 82299761,9 | 28945669,1 | 21 | 0,351710241 |
| **VAS1** | 306840869,6 | 124574715,3 | 23 | 0,405991273 |
| **NEO1** | 176264375 | 54396030,81 | 24 | 0,308604792 |
| **FINC** | 2215925000 | 918235611,6 | 24 | 0,414380275 |
| **SE6L1** | 138101909,1 | 61272407,65 | 22 | 0,443675312 |
| **RNAS1** | 705177916,7 | 276883653,5 | 24 | 0,392643682 |
| **SPIT2** | 23883277,78 | 11480440,92 | 18 | 0,480689503 |
| **NUCB1** | 191946666,7 | 40165949,64 | 24 | 0,209255781 |
| **NPDC1** | 154927444,4 | 48990333,05 | 18 | 0,316214685 |
| **UFO** | 47430166,67 | 10941778,28 | 24 | 0,230692385 |
| **ZA2G** | 2534626250 | 1655570284 | 24 | 0,653181227 |
| **PTPR2** | 74285521,74 | 25288273,5 | 23 | 0,340419949 |
| **OSTP** | 3408995833 | 1300861260 | 24 | 0,381596612 |
| **LRC4B** | 44033631,58 | 31640791,68 | 19 | 0,718559668 |
| **CO1A2** | 108624000 | 30740128,61 | 24 | 0,282995734 |
| **PTPRN** | 18876000 | 6361640,451 | 19 | 0,337022698 |
| **FA12** | 226707458,3 | 113738497,7 | 24 | 0,501697203 |
| **MUC18** | 269466250 | 73916028,73 | 24 | 0,27430533 |
| **SFRP4** | 15253271,43 | 4849861,638 | 21 | 0,317955506 |
| **FSTL4** | 26702888,89 | 17680781,8 | 18 | 0,662129924 |
| **CATL1** | 62834666,67 | 16002194,51 | 24 | 0,254671432 |
| **ATRN** | 36265583,33 | 8727429,757 | 24 | 0,24065323 |
| **FA5** | 113486583,3 | 51521952,32 | 24 | 0,453991572 |
| **FBN1** | 8592070 | 1870835,98 | 20 | 0,217739844 |
| **K1C16** | 68375230 | 75225105,64 | 20 | 1,10018066 |
| **IBP4** | 167731083,3 | 93263194,61 | 24 | 0,556028094 |
| **MOG** | 46747900 | 22495100,21 | 24 | 0,48120023 |
| **AATC** | 52945043,48 | 17530870,19 | 23 | 0,331114473 |
| **LAMC1** | 14567247,83 | 6197208,728 | 23 | 0,425420697 |
| **AGRIN** | 62877818,18 | 22035104,57 | 22 | 0,350443212 |
| **K1C9** | 3495448333 | 2485829047 | 24 | 0,711161719 |
| **PEBP4** | 175915739,1 | 49414293,38 | 23 | 0,280897512 |
| **PEBP1** | 323515833,3 | 83389311,9 | 24 | 0,2577596 |
| **FHR1** | 98072000 | 55173652,03 | 24 | 0,562583123 |
| **CBPQ** | 35492927,27 | 14488471 | 22 | 0,408207272 |
| **PRDX1** | 32545391,3 | 16885893,95 | 23 | 0,518841325 |
| **ANT3** | 4688258333 | 2127384092 | 24 | 0,45376853 |
| **NCAM1** | 1371461250 | 377588651,2 | 24 | 0,275318498 |
| **CRAC1** | 309155875 | 127940227,4 | 24 | 0,413837283 |
| **LRP1** | 32908260,87 | 9365441,708 | 23 | 0,284592423 |
| **NTRI** | 295039208,3 | 84683089,33 | 24 | 0,287023172 |
| **CNDP1** | 1173360833 | 507910850,7 | 24 | 0,432868421 |
| **CO6** | 189013291,7 | 83356578,97 | 24 | 0,441009086 |
| **CO8A** | 107941125 | 61933851,52 | 24 | 0,573774375 |
| **PENK** | 408845416,7 | 164017083,3 | 24 | 0,401171388 |
| **B2MG** | 3031462500 | 1605527301 | 24 | 0,529621363 |
| **PTPRS** | 70677045,83 | 24906152,22 | 24 | 0,352393792 |
| **TPIS** | 397028333,3 | 135744429,8 | 24 | 0,341901115 |
| **CADM1** | 205542958,3 | 51546802,42 | 24 | 0,250783597 |
| **SPON1** | 39199142,86 | 12709865,95 | 21 | 0,324238364 |
| **K2C5** | 196636826,1 | 141430123,5 | 23 | 0,719245353 |
| **NRX3A** | 155285130,4 | 49801257,21 | 23 | 0,320708474 |
| **CSTN3** | 33153523,81 | 8892335,799 | 21 | 0,268216913 |
| **A2AP** | 381846666,7 | 175726525,7 | 24 | 0,460201806 |
| **SHPS1** | 403965652,2 | 146520690,1 | 23 | 0,362705813 |
| **IBP7** | 868837083,3 | 413293070,2 | 24 | 0,475685348 |
| **THY1** | 877042916,7 | 410893445,2 | 24 | 0,468498676 |
| **CFAD** | 231515208,3 | 113254591,7 | 24 | 0,489188561 |
| **FABP5** | 31757275 | 16235692,85 | 24 | 0,511243262 |
| **LAMP2** | 321708681,8 | 130882772,8 | 22 | 0,406836309 |
| **IBP6** | 1340577500 | 335128507,1 | 24 | 0,249988163 |
| **G3P** | 57961304,35 | 38492078,67 | 23 | 0,664099594 |
| **K1C14** | 254767195,8 | 199200745,6 | 24 | 0,78189323 |
| **TYB4** | 126087260,9 | 73908006 | 23 | 0,586165529 |
| **ENPP2** | 2653908333 | 829123219,9 | 24 | 0,312415922 |
| **SIAE** | 21351578,95 | 7981764,754 | 19 | 0,373825504 |
| **RELN** | 82769457,14 | 59435290,63 | 21 | 0,718082402 |
| **FSTL1** | 102952708,3 | 37468237,53 | 24 | 0,363936395 |
| **ASPG** | 21809441,18 | 7011060,645 | 17 | 0,321469064 |
| **CFAH** | 2488483333 | 628418603 | 24 | 0,252530766 |
| **CFAB** | 2774037500 | 999129239,1 | 24 | 0,360171497 |
| **LMAN2** | 84968166,67 | 29840261,8 | 24 | 0,351193429 |
| **DCD** | 95402777,78 | 81283577,99 | 18 | 0,852004311 |
| **FAT2** | 59486909,09 | 35405407,26 | 22 | 0,59517981 |
| **PRDX2** | 655508800 | 990690656,2 | 22 | 1,511330826 |
| **TRFE** | 61003636,36 | 37618920,79 | 22 | 0,616666858 |
| **PI16** | 139760625 | 37860633,82 | 24 | 0,270896283 |
| **LYVE1** | 67887500 | 33804200,03 | 22 | 0,497944394 |
| **APOD** | 1764315417 | 1218420722 | 24 | 0,690591212 |
| **CO8B** | 50528531,82 | 38058158,99 | 22 | 0,753201362 |
| **PRIO** | 641711666,7 | 154434457,3 | 24 | 0,240660199 |
| **CO1A1** | 175485375 | 46934572,97 | 24 | 0,267455752 |
| **PGRP2** | 653780416,7 | 280554754,9 | 24 | 0,429126887 |
| **MDHC** | 45250986,96 | 20105197,78 | 23 | 0,444304072 |
| **EPCR** | 24120619,05 | 9427193,153 | 21 | 0,390835456 |
| **NRX1A** | 112359608,7 | 54510635,47 | 23 | 0,485144405 |
| **PARK7** | 30085428,57 | 16337989,43 | 21 | 0,543053239 |
| **LCAT** | 44765541,67 | 16032129,16 | 24 | 0,358135489 |
| **SODE** | 702238333,3 | 244745010,7 | 24 | 0,348521291 |
| **PPIB** | 42014142,86 | 15327986,73 | 21 | 0,364829214 |
| **FETUA** | 3000427083 | 2111840087 | 24 | 0,703846495 |
| **CNTN1** | 1617016667 | 486527159 | 24 | 0,300879496 |
| **SPRC** | 453504375 | 177137850,3 | 24 | 0,390597886 |
| **WFKN2** | 106181875 | 63648433,05 | 24 | 0,599428415 |
| **OMD** | 191081909,1 | 62770942,25 | 22 | 0,3285028 |
| **HEP2** | 181718791,7 | 83328486,11 | 24 | 0,458557342 |
| **CFAI** | 593628750 | 143745232,4 | 24 | 0,242146682 |
| **RET4** | 990875416,7 | 411003735,6 | 24 | 0,414788508 |
| **B4GA1** | 2613372500 | 1148087281 | 24 | 0,439312528 |
| **RNT2** | 216380833,3 | 61334393,21 | 24 | 0,283455758 |
| **RNAS6** | 90609136,36 | 43433795,14 | 22 | 0,479353373 |
| **CATD** | 362494000 | 138317476,7 | 24 | 0,381571769 |
| **TGON2** | 41414625 | 41539445,15 | 24 | 1,003013915 |
| **PTPRD** | 27346128,57 | 13938673,73 | 21 | 0,509712872 |
| **EFNB1** | 20519411,76 | 4989453,227 | 17 | 0,243157713 |

| **C Coefficients of variation of the analysed proteins in ALS (no missing values)** | | | | |
| --- | --- | --- | --- | --- |
| **Protein** | **Mean** | **Std** | **N** | **CV** |
| **CLUS** | 15667950000 | 3140494848 | 26 | 0,200440699 |
| **SAP3** | 840925384,6 | 241229704,8 | 26 | 0,286862199 |
| **LG3BP** | 201387692,3 | 65959717,91 | 26 | 0,327526063 |
| **ECM1** | 827839230,8 | 141913397,5 | 26 | 0,171426277 |
| **DAG1** | 596559230,8 | 105591037,1 | 26 | 0,177000089 |
| **LTBP4** | 114177769,2 | 36350546,16 | 26 | 0,318367984 |
| **PROS** | 334903076,9 | 77620713,77 | 26 | 0,23177068 |
| **GLU2B** | 61691653,85 | 17266574,62 | 26 | 0,279885099 |
| **APLP2** | 307261923,1 | 86110272,47 | 26 | 0,280250386 |
| **KPYM** | 124329692,3 | 33028046,7 | 26 | 0,265648906 |
| **PCS1N** | 914526923,1 | 216147189,2 | 26 | 0,236348634 |
| **PGCB** | 1135550769 | 307745446,2 | 26 | 0,271009852 |
| **C1QC** | 157097615,4 | 31025611,37 | 26 | 0,197492567 |
| **BIP** | 49122923,08 | 8700736,01 | 26 | 0,177121707 |
| **7B2** | 655847307,7 | 168936714,8 | 26 | 0,257585436 |
| **ENDD1** | 238442230,8 | 57702854,72 | 26 | 0,241999307 |
| **APLP1** | 3410076923 | 843447730,8 | 26 | 0,247339796 |
| **AGRL1** | 114354538,5 | 33582727,87 | 26 | 0,293672016 |
| **A4** | 2738534615 | 807625869,3 | 26 | 0,294911689 |
| **IGSF8** | 159288269,2 | 40832223,57 | 26 | 0,256341686 |
| **CD59** | 875697307,7 | 233372370,5 | 26 | 0,266498901 |
| **CALR** | 96243269,23 | 26040718,74 | 26 | 0,270571843 |
| **APOA4** | 2841111538 | 1415666028 | 26 | 0,498278934 |
| **NCAN** | 559171538,5 | 175950928,2 | 26 | 0,314663598 |
| **CADH2** | 431534230,8 | 93311621,15 | 26 | 0,216232258 |
| **ALDOA** | 75330884,62 | 25653855,42 | 26 | 0,340548973 |
| **KLK6** | 3216843077 | 999025420 | 26 | 0,310560819 |
| **CAD13** | 480084615,4 | 155113073 | 26 | 0,32309528 |
| **CO2** | 195132500 | 72482132,35 | 26 | 0,371450847 |
| **MIME** | 1925741154 | 502123453,8 | 26 | 0,260742963 |
| **FBLN3** | 939973846,2 | 209386458,4 | 26 | 0,222757749 |
| **NPC2** | 2009818462 | 555519923 | 26 | 0,276403035 |
| **VGF** | 2880532308 | 993016973,8 | 26 | 0,344733844 |
| **C1S** | 637388076,9 | 116082703,7 | 26 | 0,18212249 |
| **NELL2** | 959877307,7 | 303715094,8 | 26 | 0,316410329 |
| **THRB** | 1739792692 | 855518116,9 | 26 | 0,491735665 |
| **APOE** | 21583307692 | 6403679603 | 26 | 0,296695933 |
| **C1R** | 729235000 | 118371041 | 26 | 0,162322216 |
| **A2GL** | 214810538,5 | 120459817,9 | 26 | 0,560772385 |
| **SCG2** | 980983461,5 | 259826812,9 | 26 | 0,264863602 |
| **OPCM** | 370428846,2 | 111845918,2 | 26 | 0,301936308 |
| **NFASC** | 245228846,2 | 68617120,39 | 26 | 0,27980852 |
| **AACT** | 3158588462 | 1267623001 | 26 | 0,401325787 |
| **FAM3C** | 999401923,1 | 246983243,8 | 26 | 0,247131047 |
| **FCGBP** | 363386923,1 | 182890309,6 | 26 | 0,503293591 |
| **LY6H** | 180395423,1 | 63783750,67 | 26 | 0,353577433 |
| **LEG1** | 64098500 | 28352989,12 | 26 | 0,442334674 |
| **IBP2** | 987683846,2 | 261362236,3 | 26 | 0,264621354 |
| **C163A** | 130569153,8 | 43009984,96 | 26 | 0,329403873 |
| **CCN3** | 107826846,2 | 31126444,12 | 26 | 0,288670635 |
| **DKK3** | 11126138462 | 3451143455 | 26 | 0,3101834 |
| **SEM7A** | 345234230,8 | 141191684,8 | 26 | 0,408973596 |
| **P3IP1** | 299339230,8 | 76449652,71 | 26 | 0,255394699 |
| **CADM3** | 356475384,6 | 81418234,28 | 26 | 0,228397914 |
| **CO3A1** | 55619307,69 | 14505254,94 | 26 | 0,260795316 |
| **IC1** | 775885769,2 | 243135260,3 | 26 | 0,313364763 |
| **SCG1** | 6162880769 | 1552463040 | 26 | 0,251905415 |
| **CD14** | 754726923,1 | 217956669 | 26 | 0,288788782 |
| **SPRL1** | 3092846154 | 713406539,5 | 26 | 0,230663442 |
| **NPTX1** | 337731153,8 | 112678530,1 | 26 | 0,333633806 |
| **SAP** | 1080689346 | 391409241,7 | 26 | 0,362184788 |
| **PGBM** | 441346923,1 | 101706432,4 | 26 | 0,230445545 |
| **NEGR1** | 476078461,5 | 136745120,3 | 26 | 0,287232319 |
| **IGF2** | 496684615,4 | 188841311,9 | 26 | 0,380203667 |
| **CADM4** | 347168076,9 | 86235689,74 | 26 | 0,248397521 |
| **ACTG** | 167281807,7 | 100533735,4 | 26 | 0,600984272 |
| **CO8G** | 64278576,92 | 32831082,3 | 26 | 0,510762432 |
| **APOA2** | 1829016923 | 1251159878 | 26 | 0,684061401 |
| **CO3** | 677974230,8 | 266719360,4 | 26 | 0,393406339 |
| **NPTXR** | 771131923,1 | 231431304,8 | 26 | 0,300118952 |
| **A1BG** | 3823507692 | 1577806110 | 26 | 0,412659327 |
| **EPHA4** | 154993923,1 | 61487970,42 | 26 | 0,396712137 |
| **BTD** | 127373076,9 | 42443687,63 | 26 | 0,333223383 |
| **VTNC** | 754496923,1 | 406232799,3 | 26 | 0,538415449 |
| **LTBP2** | 45819000 | 15312842,29 | 26 | 0,334202892 |
| **CBPE** | 500928846,2 | 129424300 | 26 | 0,258368631 |
| **CD166** | 219706846,2 | 62777346,7 | 26 | 0,285732319 |
| **QSOX1** | 40876692,31 | 8174259,735 | 26 | 0,19997361 |
| **FBLN1** | 1291078462 | 249422677,7 | 26 | 0,193189403 |
| **GOLM1** | 83730346,15 | 23354892,14 | 26 | 0,278929841 |
| **ANGT** | 5044723077 | 2878641886 | 26 | 0,570624362 |
| **CYTC** | 19299557692 | 7080051376 | 26 | 0,366850448 |
| **HBB** | 22169526462 | 66162863236 | 26 | 2,984405795 |
| **NCHL1** | 1664258846 | 523794963,1 | 26 | 0,314731668 |
| **PCOC1** | 622424230,8 | 166297827,7 | 26 | 0,267177625 |
| **VTDB** | 10294692308 | 4710838696 | 26 | 0,457598785 |
| **CERU** | 6254203846 | 1802220396 | 26 | 0,288161441 |
| **ALBU** | 440193269,2 | 251985728,6 | 26 | 0,572443393 |
| **CO4B** | 13829553846 | 4697730405 | 26 | 0,339687777 |
| **ITIH1** | 307721769,2 | 208833093,9 | 26 | 0,678642575 |
| **APOH** | 6793292308 | 2913666811 | 26 | 0,428903495 |
| **NRCAM** | 2978023077 | 908610919,3 | 26 | 0,305105399 |
| **NCAM2** | 280107769,2 | 85989179,65 | 26 | 0,306986057 |
| **CA2D1** | 460748076,9 | 161679556,3 | 26 | 0,350906633 |
| **ISLR** | 52988653,85 | 6705116,172 | 26 | 0,126538715 |
| **ITIH4** | 812021923,1 | 401722674,2 | 26 | 0,494719001 |
| **TIMP1** | 493032307,7 | 115476580,2 | 26 | 0,234217065 |
| **FIBA** | 720139615,4 | 653372337,2 | 26 | 0,907285647 |
| **PLMN** | 2035107692 | 848075402,7 | 26 | 0,416722617 |
| **AMD** | 274490769,2 | 77195532,3 | 26 | 0,281231797 |
| **MEGF8** | 222268230,8 | 89457200,42 | 26 | 0,402474074 |
| **KNG1** | 838990769,2 | 446133754,9 | 26 | 0,531750493 |
| **K22E** | 1074037423 | 940046355,2 | 26 | 0,875245438 |
| **AFAM** | 1492255769 | 712611528,6 | 26 | 0,477539805 |
| **CH3L1** | 1069154615 | 711721517,3 | 26 | 0,665686241 |
| **PTGDS** | 45277192308 | 10353755763 | 26 | 0,228674863 |
| **TIMP2** | 298936153,8 | 78586968,24 | 26 | 0,262888805 |
| **PEDF** | 5289207692 | 1175107951 | 26 | 0,222170884 |
| **CNTN2** | 381446923,1 | 88270452,49 | 26 | 0,231409528 |
| **SODC** | 1504302692 | 468825316 | 26 | 0,311656237 |
| **HEMO** | 10973573077 | 3903557074 | 26 | 0,355723432 |
| **HRG** | 1219829615 | 919953233,9 | 26 | 0,754165354 |
| **SCG3** | 2285752692 | 731540189,6 | 26 | 0,320043455 |
| **CO7** | 879655384,6 | 285602923,4 | 26 | 0,324675922 |
| **ITIH2** | 340827692,3 | 195003804,6 | 26 | 0,572147772 |
| **AMBP** | 894200000 | 396542097,1 | 26 | 0,443460185 |
| **K1C10** | 3121411538 | 2948797153 | 26 | 0,944699895 |
| **CMGA** | 2974184231 | 1309018184 | 26 | 0,440126799 |
| **SE6L2** | 118586038,5 | 34271553,5 | 26 | 0,289001589 |
| **LSAMP** | 1020612308 | 233858345,1 | 26 | 0,229135337 |
| **CO6A1** | 462909615,4 | 104320704 | 26 | 0,225358689 |
| **TTHY** | 1480356154 | 562104111,5 | 26 | 0,379708701 |
| **K2C1** | 4401939231 | 3734964400 | 26 | 0,848481591 |
| **LUM** | 551799230,8 | 163795128,9 | 26 | 0,29683827 |
| **CO9** | 475556153,8 | 208258736,1 | 26 | 0,437926698 |
| **CSTN1** | 2025184615 | 718291707,1 | 26 | 0,354679619 |
| **GELS** | 7137653846 | 1359614370 | 26 | 0,190484773 |
| **CD44** | 335618538,5 | 125813091,4 | 26 | 0,374869314 |
| **CATB** | 71714884,62 | 22273565,04 | 26 | 0,310584967 |
| **TETN** | 1373492308 | 339866222 | 26 | 0,24744676 |
| **PTPRZ** | 217702692,3 | 40063336,81 | 26 | 0,184027751 |
| **CSF1R** | 217921692,3 | 114716558,9 | 26 | 0,526411839 |
| **NEO1** | 188356076,9 | 61901245 | 26 | 0,32863949 |
| **FINC** | 2068742308 | 443310471,9 | 26 | 0,214289847 |
| **RNAS1** | 762431153,8 | 278935859 | 26 | 0,365850553 |
| **NUCB1** | 201216230,8 | 50511464,55 | 26 | 0,251030766 |
| **UFO** | 49667076,92 | 11473350,42 | 26 | 0,231005147 |
| **ZA2G** | 2846789231 | 1537054886 | 26 | 0,539925777 |
| **OSTP** | 3656888462 | 1255286343 | 26 | 0,343266237 |
| **CO1A2** | 114304038,5 | 28785403,44 | 26 | 0,251831902 |
| **MUC18** | 281231153,8 | 58921911,12 | 26 | 0,209514168 |
| **ATRN** | 37863000 | 10658645,76 | 26 | 0,281505579 |
| **FA5** | 120618615,4 | 35352757,79 | 26 | 0,293095371 |
| **IBP4** | 157186923,1 | 41660996,16 | 26 | 0,265041107 |
| **K1C9** | 3164421538 | 2715895556 | 26 | 0,858259724 |
| **PEBP1** | 312963846,2 | 85448748,53 | 26 | 0,273030734 |
| **ANT3** | 4941676923 | 2131845215 | 26 | 0,431401172 |
| **NCAM1** | 1407865385 | 254978104,1 | 26 | 0,181109719 |
| **CRAC1** | 321940115,4 | 102134279,7 | 26 | 0,317246204 |
| **NTRI** | 303261923,1 | 69502953,55 | 26 | 0,22918457 |
| **CNDP1** | 1222376538 | 419073739,7 | 26 | 0,342835228 |
| **CO6** | 197868269,2 | 85844834,45 | 26 | 0,433848412 |
| **CO8A** | 102408538,5 | 43164443,1 | 26 | 0,421492619 |
| **PENK** | 423366538,5 | 118384218,2 | 26 | 0,279625826 |
| **B2MG** | 2902569231 | 997608260,9 | 26 | 0,343698352 |
| **PTPRS** | 68323884,62 | 24329798,88 | 26 | 0,356095076 |
| **TPIS** | 383571153,8 | 145329017,9 | 26 | 0,378884117 |
| **CADM1** | 210805384,6 | 59109487,38 | 26 | 0,280398375 |
| **A2AP** | 367469615,4 | 158135662,4 | 26 | 0,430336702 |
| **IBP7** | 901059230,8 | 349568279,9 | 26 | 0,38795261 |
| **THY1** | 908738076,9 | 341736086,5 | 26 | 0,376055648 |
| **CFAD** | 240024384,6 | 93300650,82 | 26 | 0,388713217 |
| **FABP5** | 33314053,85 | 21799250,9 | 26 | 0,654355996 |
| **IBP6** | 1313615385 | 385681734,5 | 26 | 0,293603241 |
| **ENPP2** | 2708276923 | 706732509,1 | 26 | 0,260952823 |
| **FSTL1** | 104968884,6 | 30614570,74 | 26 | 0,291653768 |
| **CFAH** | 2522430769 | 551233687,5 | 26 | 0,218532732 |
| **CFAB** | 2715069231 | 1172940048 | 26 | 0,432011101 |
| **LMAN2** | 86436423,08 | 26538154,87 | 26 | 0,30702514 |
| **APOD** | 1808034231 | 803034472 | 26 | 0,444147825 |
| **PRIO** | 648378461,5 | 175032213 | 26 | 0,269953775 |
| **CO1A1** | 173627038,5 | 50481673,47 | 26 | 0,290747766 |
| **PGRP2** | 662941538,5 | 307044928,6 | 26 | 0,463155362 |
| **LCAT** | 44344076,92 | 13809985,52 | 26 | 0,311427962 |
| **SODE** | 707981923,1 | 205777719,2 | 26 | 0,290653917 |
| **FETUA** | 3035800000 | 1662429456 | 26 | 0,547608359 |
| **CNTN1** | 1624816538 | 347758117,2 | 26 | 0,214029159 |
| **SPRC** | 450965384,6 | 102595697,7 | 26 | 0,227502379 |
| **WFKN2** | 107094653,8 | 40956151,24 | 26 | 0,382429466 |
| **HEP2** | 182906807,7 | 72759462,48 | 26 | 0,397795267 |
| **CFAI** | 595407692,3 | 245731630,7 | 26 | 0,412711548 |
| **RET4** | 988023846,2 | 354745781,7 | 26 | 0,359045769 |
| **B4GA1** | 2619773077 | 741878449,1 | 26 | 0,283184241 |
| **RNT2** | 216714423,1 | 62971219,34 | 26 | 0,290572351 |
| **CATD** | 362287307,7 | 95562551,27 | 26 | 0,263775598 |
| **TGON2** | 41466653,85 | 13653824,75 | 26 | 0,329272403 |

| **D Coefficients of variation of the analysed proteins in non-ALS group (no missing values)** | | | | |
| --- | --- | --- | --- | --- |
| **Protein** | **Mean** | **Std** | **N** | **CV** |
| **CLUS** | 1,0043E+10 | 3066685006 | 24 | 0,30536827 |
| **SAP3** | 505429833 | 189261133 | 24 | 0,3744558 |
| **LG3BP** | 346428333 | 125522972 | 24 | 0,3623346 |
| **ECM1** | 666598333 | 116840967 | 24 | 0,17527942 |
| **DAG1** | 489676667 | 77642302,6 | 24 | 0,15855831 |
| **LTBP4** | 78281250 | 28306759,1 | 24 | 0,36160331 |
| **PROS** | 254836667 | 75721101,3 | 24 | 0,29713582 |
| **GLU2B** | 46743625 | 10451875,7 | 24 | 0,22360003 |
| **APLP2** | 226417375 | 72387887,4 | 24 | 0,31970995 |
| **KPYM** | 91015408,3 | 35196396 | 24 | 0,3867081 |
| **PCS1N** | 718375417 | 183990015 | 24 | 0,25611959 |
| **PGCB** | 862550417 | 259256428 | 24 | 0,30056959 |
| **C1QC** | 221905667 | 92537544,4 | 24 | 0,41701299 |
| **BIP** | 40765208,3 | 8909572,05 | 24 | 0,21855824 |
| **7B2** | 508185417 | 141416299 | 24 | 0,27827697 |
| **ENDD1** | 182627500 | 61614163,4 | 24 | 0,33737615 |
| **APLP1** | 2690908333 | 714280674 | 24 | 0,26544222 |
| **AGRL1** | 84903050 | 31062812,9 | 24 | 0,36586216 |
| **A4** | 2078841667 | 632023037 | 24 | 0,30402654 |
| **IGSF8** | 121809958 | 43384984,5 | 24 | 0,35616944 |
| **CD59** | 676478667 | 219230574 | 24 | 0,32407611 |
| **CALR** | 74842375 | 23750598,8 | 24 | 0,3173416 |
| **APOA4** | 1762817500 | 1203342240 | 24 | 0,6826244 |
| **NCAN** | 424797042 | 150883933 | 24 | 0,35519064 |
| **CADH2** | 357647917 | 90116820,9 | 24 | 0,25197077 |
| **ALDOA** | 99771583,3 | 35678689,7 | 24 | 0,35760372 |
| **KLK6** | 2496860833 | 800922854 | 24 | 0,32077192 |
| **CAD13** | 375944583 | 106507761 | 24 | 0,28330708 |
| **CO2** | 275859583 | 129845207 | 24 | 0,47069312 |
| **MIME** | 1560606250 | 455019158 | 24 | 0,29156564 |
| **FBLN3** | 767101167 | 250341758 | 24 | 0,32634777 |
| **NPC2** | 1663491667 | 331497017 | 24 | 0,19927783 |
| **VGF** | 2132979167 | 1020178317 | 24 | 0,47828799 |
| **C1S** | 772244167 | 234036933 | 24 | 0,3030608 |
| **NELL2** | 748767500 | 268451364 | 24 | 0,35852433 |
| **THRB** | 1171229167 | 686716962 | 24 | 0,5863216 |
| **APOE** | 1,705E+10 | 6115726770 | 24 | 0,35868857 |
| **C1R** | 888446250 | 294812172 | 24 | 0,33182893 |
| **A2GL** | 344081833 | 230226222 | 24 | 0,6691031 |
| **SCG2** | 798176667 | 253560530 | 24 | 0,3176747 |
| **OPCM** | 291859500 | 110306303 | 24 | 0,37794317 |
| **NFASC** | 199127475 | 61265302,7 | 24 | 0,30766876 |
| **AACT** | 4254350000 | 1806976050 | 24 | 0,42473611 |
| **FAM3C** | 827699167 | 241795966 | 24 | 0,29213025 |
| **FCGBP** | 865529583 | 1028523715 | 24 | 1,18831723 |
| **LY6H** | 140149833 | 53031070,5 | 24 | 0,3783884 |
| **LEG1** | 48208750 | 18677098,4 | 24 | 0,38742134 |
| **IBP2** | 821864167 | 245506474 | 24 | 0,29871904 |
| **C163A** | 193207667 | 135843682 | 24 | 0,70309675 |
| **CCN3** | 88664125 | 29697040,3 | 24 | 0,33493863 |
| **DKK3** | 9369591667 | 1902425989 | 24 | 0,20304257 |
| **SEM7A** | 263453958 | 121735467 | 24 | 0,46207492 |
| **P3IP1** | 257307917 | 60033509,2 | 24 | 0,23331388 |
| **CADM3** | 301084500 | 101031023 | 24 | 0,33555704 |
| **CO3A1** | 46832666,7 | 14614235,1 | 24 | 0,31205217 |
| **IC1** | 937131250 | 302445303 | 24 | 0,32273527 |
| **SCG1** | 5223470833 | 1685170526 | 24 | 0,32261509 |
| **CD14** | 942184167 | 409924506 | 24 | 0,43507896 |
| **SPRL1** | 2680141667 | 736040184 | 24 | 0,27462734 |
| **NPTX1** | 279771708 | 97451712,1 | 24 | 0,34832583 |
| **SAP** | 898398750 | 258762471 | 24 | 0,2880263 |
| **PGBM** | 386625000 | 99835981,8 | 24 | 0,25822433 |
| **NEGR1** | 401524667 | 139228928 | 24 | 0,34675062 |
| **IGF2** | 407714583 | 134572026 | 24 | 0,33006429 |
| **CADM4** | 301133750 | 85973375,6 | 24 | 0,28549897 |
| **ACTG** | 235753833 | 156962444 | 24 | 0,66578957 |
| **CO8G** | 85255416,7 | 46802398,8 | 24 | 0,54896686 |
| **APOA2** | 1284167917 | 793933357 | 24 | 0,61824731 |
| **CO3** | 551311667 | 229200930 | 24 | 0,41573749 |
| **NPTXR** | 647063667 | 275348381 | 24 | 0,42553522 |
| **A1BG** | 4673541667 | 1980091895 | 24 | 0,42368123 |
| **EPHA4** | 127706413 | 54187692,2 | 24 | 0,42431458 |
| **BTD** | 148481292 | 47806596,3 | 24 | 0,3219705 |
| **VTNC** | 593455000 | 265956002 | 24 | 0,44814856 |
| **LTBP2** | 38261625 | 17526717,4 | 24 | 0,45807562 |
| **CBPE** | 435687083 | 155001074 | 24 | 0,35576238 |
| **CD166** | 194263000 | 49098553,5 | 24 | 0,25274269 |
| **QSOX1** | 44682416,7 | 8938144,64 | 24 | 0,20003718 |
| **FBLN1** | 1165843333 | 321828244 | 24 | 0,27604759 |
| **GOLM1** | 73276083,3 | 25366766,1 | 24 | 0,3461807 |
| **ANGT** | 6147108333 | 2212998773 | 24 | 0,36000647 |
| **CYTC** | 1,6705E+10 | 5111396830 | 24 | 0,30598112 |
| **HBB** | 5,4541E+10 | 9,0844E+10 | 24 | 1,66561189 |
| **NCHL1** | 1451475833 | 514333023 | 24 | 0,35435177 |
| **PCOC1** | 555002083 | 163218180 | 24 | 0,29408571 |
| **VTDB** | 8652075000 | 3254565740 | 24 | 0,37616014 |
| **CERU** | 7392454167 | 3637766584 | 24 | 0,49209187 |
| **ALBU** | 625982375 | 616247884 | 24 | 0,98444926 |
| **CO4B** | 1,5912E+10 | 5758896706 | 24 | 0,36192076 |
| **ITIH1** | 457168458 | 498150814 | 24 | 1,08964388 |
| **APOH** | 5811683333 | 1926309759 | 24 | 0,3314547 |
| **NRCAM** | 2634830000 | 897263064 | 24 | 0,34053926 |
| **NCAM2** | 248104583 | 89080953,3 | 24 | 0,35904598 |
| **CA2D1** | 400437167 | 168471739 | 24 | 0,42071954 |
| **ISLR** | 49105958,3 | 13797648,6 | 24 | 0,28097708 |
| **ITIH4** | 1002423333 | 635994983 | 24 | 0,63445748 |
| **TIMP1** | 620017500 | 495118374 | 24 | 0,79855548 |
| **FIBA** | 1619917500 | 3545981194 | 24 | 2,18898876 |
| **PLMN** | 1757008750 | 701749609 | 24 | 0,39940018 |
| **AMD** | 243869292 | 96853855,5 | 24 | 0,39715478 |
| **MEGF8** | 192947458 | 77204785,5 | 24 | 0,40013373 |
| **KNG1** | 710494583 | 295514568 | 24 | 0,41592797 |
| **K22E** | 1406476167 | 1041825451 | 24 | 0,74073452 |
| **AFAM** | 1283815833 | 502431533 | 24 | 0,39135795 |
| **CH3L1** | 1440008500 | 1445300074 | 24 | 1,00367468 |
| **PTGDS** | 4,8703E+10 | 1,0693E+10 | 24 | 0,21956311 |
| **TIMP2** | 271632917 | 89347167,1 | 24 | 0,32892614 |
| **PEDF** | 5724433333 | 1540412138 | 24 | 0,26909426 |
| **CNTN2** | 346446000 | 135316530 | 24 | 0,39058477 |
| **SODC** | 1642645000 | 436114894 | 24 | 0,26549552 |
| **HEMO** | 1,2177E+10 | 4299186747 | 24 | 0,35306593 |
| **HRG** | 1462845833 | 744961655 | 24 | 0,50925507 |
| **SCG3** | 2080092500 | 703593949 | 24 | 0,33825128 |
| **CO7** | 986452500 | 454507042 | 24 | 0,46074904 |
| **ITIH2** | 427009417 | 392594946 | 24 | 0,91940583 |
| **AMBP** | 796940000 | 287179481 | 24 | 0,3603527 |
| **K1C10** | 3905927917 | 2731133716 | 24 | 0,69922789 |
| **CMGA** | 2609429583 | 1345941981 | 24 | 0,51579931 |
| **SE6L2** | 106947292 | 49696917 | 24 | 0,46468607 |
| **LSAMP** | 958085000 | 222723585 | 24 | 0,23246746 |
| **CO6A1** | 429976667 | 135922624 | 24 | 0,31611628 |
| **TTHY** | 1639228333 | 651045883 | 24 | 0,39716608 |
| **K2C1** | 5316275417 | 3489504980 | 24 | 0,65638153 |
| **LUM** | 509451667 | 180301941 | 24 | 0,35391373 |
| **CO9** | 426836667 | 188091214 | 24 | 0,44066321 |
| **CSTN1** | 1860897500 | 639462142 | 24 | 0,34363104 |
| **GELS** | 6847004167 | 1030957520 | 24 | 0,1505706 |
| **CD44** | 307586833 | 116396533 | 24 | 0,37841845 |
| **CATB** | 80868291,7 | 53338971,4 | 24 | 0,65957831 |
| **TETN** | 1289072917 | 417368204 | 24 | 0,32377393 |
| **PTPRZ** | 209023333 | 39471077,5 | 24 | 0,18883575 |
| **CSF1R** | 195497708 | 93213255,4 | 24 | 0,47679973 |
| **NEO1** | 176264375 | 54396030,8 | 24 | 0,30860479 |
| **FINC** | 2215925000 | 918235612 | 24 | 0,41438028 |
| **RNAS1** | 705177917 | 276883654 | 24 | 0,39264368 |
| **NUCB1** | 191946667 | 40165949,6 | 24 | 0,20925578 |
| **UFO** | 47430166,7 | 10941778,3 | 24 | 0,23069239 |
| **ZA2G** | 2534626250 | 1655570284 | 24 | 0,65318123 |
| **OSTP** | 3408995833 | 1300861260 | 24 | 0,38159661 |
| **CO1A2** | 108624000 | 30740128,6 | 24 | 0,28299573 |
| **MUC18** | 269466250 | 73916028,7 | 24 | 0,27430533 |
| **ATRN** | 36265583,3 | 8727429,76 | 24 | 0,24065323 |
| **FA5** | 113486583 | 51521952,3 | 24 | 0,45399157 |
| **IBP4** | 167731083 | 93263194,6 | 24 | 0,55602809 |
| **K1C9** | 3495448333 | 2485829047 | 24 | 0,71116172 |
| **PEBP1** | 323515833 | 83389311,9 | 24 | 0,2577596 |
| **ANT3** | 4688258333 | 2127384092 | 24 | 0,45376853 |
| **NCAM1** | 1371461250 | 377588651 | 24 | 0,2753185 |
| **CRAC1** | 309155875 | 127940227 | 24 | 0,41383728 |
| **NTRI** | 295039208 | 84683089,3 | 24 | 0,28702317 |
| **CNDP1** | 1173360833 | 507910851 | 24 | 0,43286842 |
| **CO6** | 189013292 | 83356579 | 24 | 0,44100909 |
| **CO8A** | 107941125 | 61933851,5 | 24 | 0,57377438 |
| **PENK** | 408845417 | 164017083 | 24 | 0,40117139 |
| **B2MG** | 3031462500 | 1605527301 | 24 | 0,52962136 |
| **PTPRS** | 70677045,8 | 24906152,2 | 24 | 0,35239379 |
| **TPIS** | 397028333 | 135744430 | 24 | 0,34190112 |
| **CADM1** | 205542958 | 51546802,4 | 24 | 0,2507836 |
| **A2AP** | 381846667 | 175726526 | 24 | 0,46020181 |
| **IBP7** | 868837083 | 413293070 | 24 | 0,47568535 |
| **THY1** | 877042917 | 410893445 | 24 | 0,46849868 |
| **CFAD** | 231515208 | 113254592 | 24 | 0,48918856 |
| **FABP5** | 31757275 | 16235692,9 | 24 | 0,51124326 |
| **IBP6** | 1340577500 | 335128507 | 24 | 0,24998816 |
| **ENPP2** | 2653908333 | 829123220 | 24 | 0,31241592 |
| **FSTL1** | 102952708 | 37468237,5 | 24 | 0,3639364 |
| **CFAH** | 2488483333 | 628418603 | 24 | 0,25253077 |
| **CFAB** | 2774037500 | 999129239 | 24 | 0,3601715 |
| **LMAN2** | 84968166,7 | 29840261,8 | 24 | 0,35119343 |
| **APOD** | 1764315417 | 1218420722 | 24 | 0,69059121 |
| **PRIO** | 641711667 | 154434457 | 24 | 0,2406602 |
| **CO1A1** | 175485375 | 46934573 | 24 | 0,26745575 |
| **PGRP2** | 653780417 | 280554755 | 24 | 0,42912689 |
| **LCAT** | 44765541,7 | 16032129,2 | 24 | 0,35813549 |
| **SODE** | 702238333 | 244745011 | 24 | 0,34852129 |
| **FETUA** | 3000427083 | 2111840087 | 24 | 0,7038465 |
| **CNTN1** | 1617016667 | 486527159 | 24 | 0,3008795 |
| **SPRC** | 453504375 | 177137850 | 24 | 0,39059789 |
| **WFKN2** | 106181875 | 63648433,1 | 24 | 0,59942842 |
| **HEP2** | 181718792 | 83328486,1 | 24 | 0,45855734 |
| **CFAI** | 593628750 | 143745232 | 24 | 0,24214668 |
| **RET4** | 990875417 | 411003736 | 24 | 0,41478851 |
| **B4GA1** | 2613372500 | 1148087281 | 24 | 0,43931253 |
| **RNT2** | 216380833 | 61334393,2 | 24 | 0,28345576 |
| **CATD** | 362494000 | 138317477 | 24 | 0,38157177 |
| **TGON2** | 41414625 | 41539445,2 | 24 | 1,00301392 |

**Supplementary Figure 1**. Boxplots for significant (p<0.05) differences between patients and controls, allowing for 20% missing values.

**Supplementary Figure 2**. Boxplots for significant (p<0.05) differences between patients and controls, with no missing values.
